# Non-invasive skin sampling of tryptophan/kynurenine ratio in vitro towards a skin cancer biomarker

**DOI:** 10.1038/s41598-020-79903-w

**Published:** 2021-01-12

**Authors:** Skaidre Jankovskaja, Johan Engblom, Melinda Rezeli, György Marko-Varga, Tautgirdas Ruzgas, Sebastian Björklund

**Affiliations:** 1 Department of Biomedical Science, Faculty of Health and Society, Malmö University, 205 06 Malmö, Sweden; 2 Biofilms-Research Center for Biointerfaces, Malmö University, 205 06 Malmö, Sweden; 3 Clinical Protein Science and Imaging, Department of Biomedical Engineering, Lund University, Lund, Sweden

**Keywords:** Skin cancer, Tumour biomarkers, Skin cancer, Biophysical chemistry, Biomarkers, Biophysics

## Abstract

The tryptophan to kynurenine ratio (Trp/Kyn) has been proposed as a cancer biomarker. Non-invasive topical sampling of Trp/Kyn can therefore serve as a promising concept for skin cancer diagnostics. By performing in vitro pig skin permeability studies, we conclude that non-invasive topical sampling of Trp and Kyn is feasible. We explore the influence of different experimental conditions, which are relevant for the clinical in vivo setting, such as pH variations, sampling time, and microbial degradation of Trp and Kyn. The permeabilities of Trp and Kyn are overall similar. However, the permeated Trp/Kyn ratio is generally higher than unity due to endogenous Trp, which should be taken into account to obtain a non-biased Trp/Kyn ratio accurately reflecting systemic concentrations. Additionally, prolonged sampling time is associated with bacterial Trp and Kyn degradation and should be considered in a clinical setting. Finally, the experimental results are supported by the four permeation pathways model, predicting that the hydrophilic Trp and Kyn molecules mainly permeate through lipid defects (i.e., the porous pathway). However, the hydrophobic indole ring of Trp is suggested to result in a small but noticeable relative increase of Trp diffusion via pathways across the SC lipid lamellae, while the shunt pathway is proposed to slightly favor permeation of Kyn relative to Trp.

## Introduction

Skin cancer is a frequently diagnosed malignancy worldwide. Consequently, numerous attempts have been made to reduce skin cancer risk factors, such as avoidance of ultraviolet radiation exposure. However, skin cancer incidence continues to rise and improved diagnostic biomarkers are therefore of increasing importance. Early stage detection of skin cancer is crucial since it increases the probability of successful treatment to avoid cancer progression and eventual morbidity and mortality^1^. To date, visual inspection followed by biopsy is the golden standard of skin cancer diagnosis^1^. A major drawback of this approach is its inherently low accuracy, in some cases with an accuracy rate as low as 22%^2^. This is particularly problematic considering that it is a relatively expensive and invasive procedure that can cause complications such as infections around the punctured/damaged area^3^. To limit the magnitude of these shortcomings, it is clear that complementary skin cancer diagnostic methods are highly relevant to develop as support for the decision of taking a biopsy or not. In fact, most skin cancers are curable if detected early enough. Therefore, detection of cancer biomarkers by non-invasive methods in suspected cancer lesions would be of great benefit.

Based on improved understanding of the molecular pathways underlying skin cancer pathogenesis, new hypotheses have been formulated for skin cancer biomarkers. Non-invasive topical sampling and analysis of chemical compounds, originating from the skin organ, represents an attractive option for skin cancer diagnosis due to its expected low cost and easy access to the skin sampling site. This strategy could be utilized in several stages during the disease progression and include, for example, detection of changes in quantity and composition of lipids, proteins, nucleic acids, and small biomolecules^4^. However, despite an increasing number of molecules recognized as skin cancer biomarkers, e.g. cytokines such as interleukin 6 (IL-6) or interferon gamma (IFN-γ)^5^, enzyme indoleamine-2,3-dioxygenase^6^, v-raf murine sarcoma viral oncogene homologue B1 (BRAF) gene mutations^7^, their applications are limited in terms of non-invasive diagnostics. The majority of these biomarkers are macromolecules (e.g., proteins with high molecular weight), which reside in the viable epidermis. This means that they will not permeate the skin barrier and therefore not be available via non-invasive topical sampling from the skin surface^8^. The challenge for molecules to penetrate the skin organ is related to the outermost skin layer, the stratum corneum (SC), which can be envisaged as a brick wall composed of corneocytes (bricks) embedded in the continuous matrix of lipids (mortar)^9^. The lipophilic nature of the SC greatly restricts the permeation of hydrophilic and high molecular weight substances (> 500 Da) through intact skin^8^. Thus, for non-invasive topical skin cancer diagnostics we should look for biomarkers of low molecular weight (< 500 Da) with a realistic ability to partition and permeate through the SC barrier.

Recent reports have shown that the established cancer biomarker indoleamine-2,3-dioxygenase 1 (IDO1), which is overexpressed in cancer microenvironments, increases the conversion of tryptophan (Trp) to kynurenine (Kyn)^10–12^. This alteration in Trp metabolism initiates/facilitates cancer development and progression. However, the precise mechanism of the Trp and Kyn involvement in cancer development is not yet clarified. One hypothesis is that the local depletion of Trp in the tumor microenvironment induces T-cell starvation, leading to reduced efficiency of the immune system to clear cancer cells^13^. Another hypothesis is that the build-up of Kyn, which is a downstream catabolite of Trp, facilitates cancer immune escape by initiating several downstream processes, e.g. reprogramming T-helper cells into T-regulatory cells^13,14^. Both of these mechanisms most likely result in a decrease of the Trp/Kyn ratio at the tumor site. This raises the question if the Trp/Kyn ratio can be regarded as a general biomarker for cancer. In particular for various skin cancers, since both Trp and Kyn are low molecular weight substances that are expected to diffuse relatively unrestricted across the skin barrier tissue and thus have sufficiently high skin surface concentrations for non-invasive topical sampling of the Trp/Kyn ratio. In this context, it is crucial to characterize the skin permeabilities of Trp and Kyn at different conditions in order to evaluate if the Trp/Kyn ratio can be monitored non-invasively from samples collected from the skin surface.

With this as background, the objective of this study was to assess the feasibility of non-invasive topical monitoring of the Trp/Kyn ratio, as a potential skin cancer biomarker, by performing a comprehensive series of in vitro experiments. In particular, the series of experiments was designed to address the following research issues:

1. The effect of pH on Trp and Kyn skin permeability and the Trp/Kyn ratio,
2. The influence of endogenous Trp on the Trp/Kyn ratio,
3. Skin membrane partitioning of Trp and Kyn as a function of pH,
4. The influence of microbial degradation of Trp and Kyn on the sampled Trp/Kyn ratio,
5. Comparison of experimental and theoretical Trp and Kyn skin permeability coefficients.

The relevance for these topics is briefly motivated in the following. Firstly, it is expected that the pH of the aqueous microenvironment in the skin can vary between pH 4.5–6.0^15–17^. In the case of a defective skin barrier, such as an open wound, the pH of the aqueous microenvironment can be significantly elevated to values as high as 9.0^18^. Thus, it is clear that pH fluctuations, during in vivo topical skin sampling in clinical settings, should be expected, which in turn can influence the skin permeability of Trp and Kyn, including the Trp/Kyn ratio. Secondly, since Trp is naturally present in the SC as a component of the natural moisturizing factor (NMF)^19^, it is important to consider the influence of endogenous Trp on the sampled Trp/Kyn ratio. Further, to improve the understanding of the Trp and Kyn skin permeability characteristics, including their partitioning behavior between the buffer medium in contact with the skin membrane and its interior, as a function of pH, it is advantageous to perform a mass balance analysis. Next, it is not unrealistic to expect that microbial degradation of Trp and Kyn can occur in an in vivo clinical setting, which, if present, undoubtedly would influence the outcome of the sampled Trp/Kyn ratio. In fact, this topic is seldom considered when performing in vitro permeation studies (for example, this issue is not considered in the OECD guidelines for skin absorption in vitro). Finally, the experimental results should be evaluated within an appropriate theoretical framework to improve the understanding of the skin permeability characteristics, with the ultimate aim to predict the dominating transport pathways across the skin barrier for the investigated biomarkers. To examine this issue, the four permeation pathways model was employed in the present work^23^.

Taken together, the present series of in vitro experiments allows for a detailed analysis of the Trp and Kyn skin permeability characteristics, as shown by the results, and provide several important conclusions that should be considered before initiating in vivo studies and establishing clinical procedures of non-invasive sampling of the Trp/Kyn ratio from the skin surface as a skin cancer biomarker.

## Materials and methods

### Materials

Trp (l-tryptophan) and Kyn (l-kynurenine) were purchased from Sigma-Aldrich (St. Louis, MO, USA) at reagent grade > 98% (HPLC). A fresh stock solution, containing both Trp and Kyn at concentrations of 20 mM, was prepared in Milli-Q water the same day as a permeation experiment was initiated. The fresh stock solution was kept at − 20 °C for a maximum of 2 days and used to prepare calibration solutions for HPLC–UV analysis.

Methanol of HPLC gradient grade was obtained from VWR International (Fontenay-sous-Bois, France). Formic acid (> 99%) was purchased from Merck (Darmstadt, Germany). NaN_3_, 30% HCl, and Na_2_HPO_4_·2H_2_O were obtained from Sigma-Aldrich (St. Louis, MO, USA). KCl, NaCl, while KH_2_PO_4_ and NaH_2_HPO_4_·H_2_O were purchased from Merck (Darmstadt, Germany). Phosphate buffered saline (PBS, pH = 7.4) was prepared from Milli-Q water with concentrations of 130.9 mM NaCl, 5.1 mM Na_2_HPO_4_·2H_2_O, and 1.5 mM KH_2_PO_4_.

### Split-thickness skin membranes

Skin from pig ears was used as skin model in this study. The pig ears were obtained from local abattoir (Strömbäcks Gårdsslakteri, Illstorp, Sweden) and kept at − 80 °C until their usage (maximum storage time was a few months). To prepare skin membranes, defrosted pig ears were cleaned with cold water and cut into strips with a scalpel. The strips were shaved and approximately 0.5 mm thick skin membranes were sliced with a dermatome (TCM 3000 BL, Nouvag, Konstanz, Germany). The resulting skin strips were punched out to form circular membranes (ca. 16 mm diameter) and kept at − 20 °C on a filter paper soaked with PBS until their usage (maximum storage time was two months). Skin membranes used for permeation studies were randomly selected from ears from different pigs.

### Skin membrane electrical resistance

The integrity of the skin membranes was evaluated by measuring the electrical impedance before and after each permeation experiment. Measurements were conducted with the skin membrane mounted in a Franz cell equipped with four electrodes: sensing, working, reference, and counter. Briefly, the impedance measurements were carried out with a potentiostat from Ivium Technologies (Eindhoven, Netherlands) within a frequency range between 1 and 10^6^ Hz and a maximum of the voltage amplitude of 10 mV. The skin membrane resistance was determined by fitting the impedance versus frequency data to an equivalent circuit using the Ivium software. For this, an equivalent circuit comprised of a resistor, for solution resistance, connected in series with the skin membrane impedance was used. The skin membrane impedance was modelled by a parallel combination of a resistor for the skin membrane resistance, $R_{mem}$, and a so-called constant phase element, *CPE*, which here represents the capacitive properties of the skin membrane^20–22^. A more detailed description of Franz cell equipped with four electrodes for measurement of skin membrane resistance can be found elsewhere^20^.

### Franz cell permeability experiments

**Figure 1:**
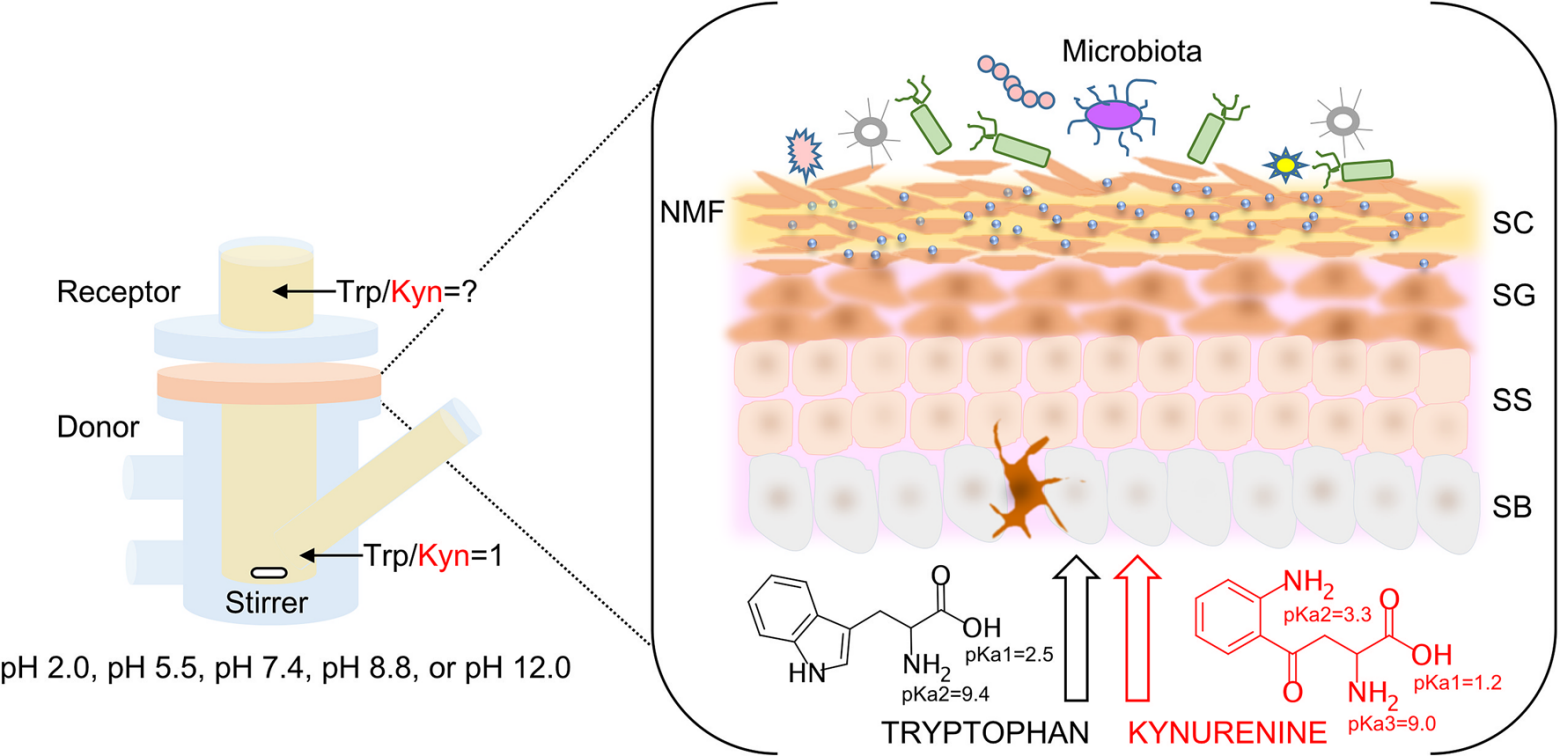
Schematic representation of the in vitro Franz cell setup. *NMF *natural moisturizing factor, *SC *stratum corneum, *SG *stratum granulosum, *SS *stratum spinosum, *SB *stratum basale.

The permeabilities of Trp and Kyn trough skin membranes were determined by using a Franz cell setup (Fig. 1) (from PermeGear Inc.; d = 9 mm, V = 6 mL) with effective diffusional area of 0.64 cm^2^. The skin membrane was mounted between the two chambers with the dermal side facing down. The donor chamber (here the lower chamber of the Franz cell) was filled with 5.4 mL of PBS containing 15 mM of NaN_3_. In some experiments, the pH of the PBS was adjusted to 5.5, 7.4 or 8.8, while 131 mM NaCl solution was used for the cases when the pH was adjusted to 2.0 and 12.0. The receptor chamber (here the upper chamber of the Franz cell) was filled with 0.6 mL of the corresponding donor solution. The skin membrane, mounted in the Franz cell, was left to equilibrate for about 30 min and then an electrical impedance measurement was conducted as described above. After that, the PBS solution from the upper receptor chamber was replaced with fresh solution. Then, equal aliquots of Trp and of Kyn stock solution (20 mM) was mixed before injecting 600 µL of this mixture into the center of the lower donor chamber with a thin needle. This resulted in a total volume of 6 mL in the lower donor chamber with 1 mM concentrations of both Trp and Kyn. In the negative control experiments, no Trp or Kyn was added. The solution containing the analytes was continuously mixed with a magnetic stirrer and the temperature of the donor chamber was maintained constant by a water bath heating system set at 32 °C. Samples of 500 µL were withdrawn from the upper receptor chamber and replaced with an identical volume of fresh solution at determined time points of 0.5 h, 1 h, 2 h, 4 h, 8 h, 22 h, 26 h and 45 h. The collected aliquots were placed in 1.5 mL vials, dried by evaporation with a Genevac system at 35 °C (EZ-2 Plus Evaporating System, Genevac LTD., England), and kept at + 4 °C for a maximum time of 2 days until analyzed by HPLC–UV. Prior to analysis, the dried samples were resuspended in 50 µL of MilliQ water. The permeability experiments without addition of NaN_3_ were performed in six replicates for each pH value, while the experiments with addition of NaN_3_ were performed in triplicates. Control experiments, with the Franz cell set-up, with no added Trp and Kyn in the lower donor chamber, were done in triplicates (pH of 7.4). Further, the corresponding skin membranes from these control experiments were used to determine the presence of endogenous Trp and Kyn by the extraction procedure described below.

To evaluate the results, the permeability coefficient ($K_{p}$) was calculated from the cumulative permeated mass over time (${\text{d}}Q/{\text{d}}t$), normalized by the effective permeation area, ($A =$ 0.64 cm^2^) according to the following equation:

1 $$K_{p} = \frac{1}{A\Delta C}{ }\frac{{{\text{d}}Q}}{{{\text{d}}t}} = \frac{{J_{ss} }}{\Delta C}$$

In Eq. (1), $\Delta C$ represents the concentration difference between the diffusion cell compartments, which was concluded to be constant at 1 mM within the limit of uncertainty. The steady state flux ($J_{ss}$) was estimated from the cumulative permeation profiles by linear fits of three data points, corresponding to 2, 4 and 8 h. In some cases, the amount of Trp or Kyn was not quantifiable in the collected fractions, in particular for fractions collected after 30 min, 1 h, or 2 h. In these cases, the amount Trp or Kyn was assumed to be equal to zero.

### Mass balance analysis

A mass balance evaluation was performed to characterize how Trp and Kyn are distributed between the receptor chamber, the donor chamber, and the skin membrane. Hence, at the end of the experiment (after 45 h), the skin membrane was removed from the Franz cell and rinsed by immersing the membrane into 20 mL of PBS solution for about 10 s. After that, the skin membrane was placed in a glass vial containing 5 mL of 131 mM NaCl (pH of 12.0) and sonicated for 1 h for extraction of Trp and Kyn. For the calculations of the results from these experiments, only the skin area exposed to Trp and Kyn solution was taken into account, corresponding to 0.64 cm^2^. However, in the control experiments, where no Trp and Kyn were added, the total area of the skin membrane was used (2.0 cm^2^). Next, the solution was collected in a centrifuge tube, while the described extraction procedure was repeated two more times. Finally, the three extraction solutions were dried by evaporation by a Genevac system at 35 °C. The obtained samples were resuspended in 500 µL of MilliQ water and syringe filtered (13 mm, w/0.2 μm PTFE membrane, VWR International, USA) prior to the HPLC analysis. The amount of Trp and Kyn remaining in the lower donor chamber was estimated by diluting the sample 10 times and directly measuring it with HPLC–UV.

### HPLC–UV analysis of tryptophan and kynurenine

The HPLC–UV analysis of Trp and Kyn was performed using an Agilent system (Agilent 1100 Series, Germany) equipped with a G1312A binary pump, G1322A in-line degasser, G1316A column oven, G1313A autosampler, and G1315 diode array detector. The separation was performed with a Kromasil C18 analytical column (250 mm × 4.6 mm ID) with particle size of 5 µm (AkzoNobel, Bellefonte, USA) under gradient elution created from solvent A (10 mM of NaH_2_PO_4_ at pH of 2.8) and solvent B (100% of methanol) at a flow rate of 0.9 mL/min and 40 °C. The gradient profile was as follows: 20% B was held for 7 min, then increased to 95% B over 2 min and held at 95% B for 4 min; then solvent B was decreased to 20% B in 0.1 min and kept at 20% B for 2.9 min.

Trp was monitored at 280 nm and Kyn at 360 nm of UV wavelengths. The peaks of Kyn and Trp were identified by comparison of their retention times with the established retention times from calibration solutions. The concentrations of Trp and Kyn were calculated by integration of the peak areas using an OpenLAB software (Lab Advisor Basic Software; Agilent; Germany). All integrated peaks were inspected manually. The unknown concentrations of Trp and Kyn were calculated based on calibration solutions in the concentration range from 1.6 μM to 50 μM (R^2^ > 0.99).

### Calculation of permeability coefficients based on the four permeation pathways theory

The theoretical fluxes of Trp and Kyn can be calculated based on the four permeation pathways model according to the following equation:

2 $$J_{ss} = K_{p} \times \Delta C = \left[ {K_{p}^{fv} + K_{p}^{lateral} + K_{p}^{shunt} + K_{p}^{pore} } \right] \times \Delta C$$

where $K_{p}^{fv}$, $K_{p}^{lateral}$, $K_{p}^{shunt}$, and $K_{p}^{pore}$ represents the permeability constants accounting for diffusion via the free-volume pathway through lipid bilayers, lateral diffusion along lipid bilayers, diffusion through shunt pathways, and finally diffusion through pores created by imperfections in the multilamellar lipid matrix^23^. A detailed description of these calculations, including the physicochemical input parameters for Trp and Kyn (see Table S4), is presented in the Supplementary Information. In brief, the free-volume and lateral diffusion via the SC lipid domains are modelled based on the size of the permeants and the distribution coefficients between octanol and water, while the shunt pathway, which is expected to primarily be important for high molecular weight hydrophilic molecules, is assumed to be equal for both permeants. Finally, the porous pathway is modelled based on experimentally determined values of skin membrane resistance and the electrolyte ion permeability coefficients. The latter pathway is envisaged to occur via dynamical (non-static) imperfections in the lipid matrix, which can support water-filled pores that may act as pathways for hydrophilic small molecules, such as Trp and Kyn.

### Statistical analysis

Statistical analysis was performed using one-way ANOVA test. A *p*-value less than 0.05 was considered to be statistically significant. The results are consistently presented as mean values ± standard errors of the mean (SEM).

## Results and discussion

To enable sampling and analysis of the potential skin cancer biomarker, i.e., the Trp/Kyn ratio, from the skin surface, it is required to characterize how the Trp/Kyn ratio on the surface of the skin correlates with the ratio existing in the viable epidermis. The SC skin barrier restricts permeation of molecules depending on their physicochemical properties, such as molecular weight, chemical structure and functional groups, partition coefficient (i.e., lipophilic characteristics), electrical charge characteristics, etc. Thus, it is clear that the physicochemical properties of the permeant will influence the diffusion pathway across the SC and this can be modelled, for example, by the four permeation pathways model, which was used in this study^23^. It is also important to note that the SC is not an inert membrane. In contrast, the properties of the skin barrier can be greatly influenced by the external environment, such as hydration^20,24^, temperature^25^, presence of common excipients in skin formulations^21,26^ or chemical penetration enhancers^27^.

The starting point of the present work was to investigate the effect of pH on the skin permeability of Trp and Kyn, which is important for two main reasons. First, the pH influence both the electrical charge status of the permeants, and thus their distribution coefficients (logD values), and also the properties of the skin barrier. Secondly, the pH value may vary significantly between healthy and diseased skin states and also in conditions with or without occlusion^15,18,28^. In the following part of this study we focused on the fact that in vivo sampling of Trp and Kyn may involve a situation where degradation of Trp and Kyn by skin microbiota is likely to occur. To approach this topic, the sampling time of these permeants was assessed by running experiments extended in time. Finally, due to the fact that Trp is a natural constituent of the NMF in the SC^29^, one needs to consider how the endogenous pool of Trp influences the sampled Trp/Kyn ratio with the final aim to separate this fraction from the Trp originating from the cancerous epidermal site. These question have been addressed by performing in vitro permeability measurements and rationalizing the obtained results in the frame of the four permeation pathways theory^23^.

### The effect of pH on Trp and Kyn skin permeability and the Trp/Kyn ratio

**Figure 2:**
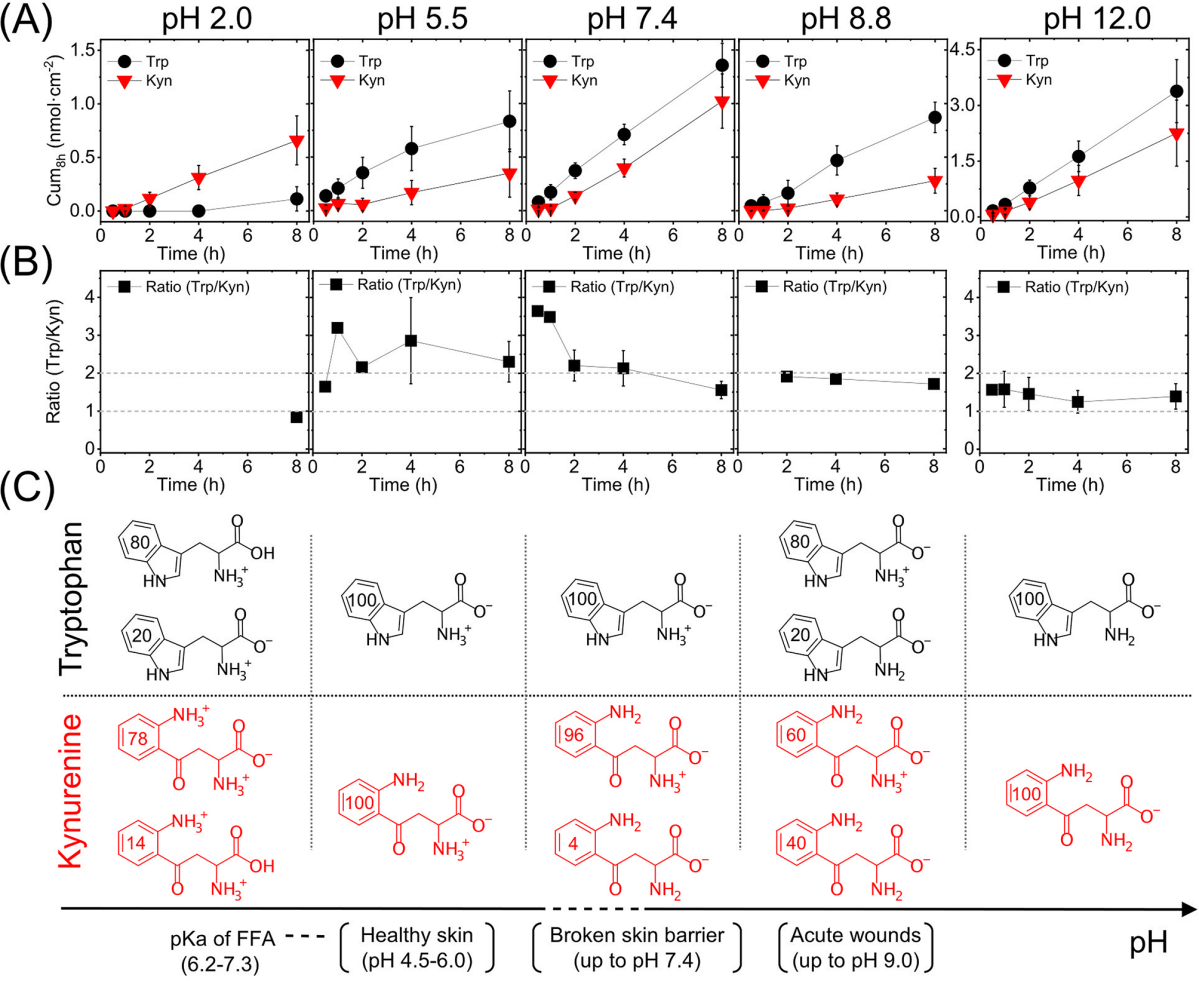
(**A**) Cumulative permeated amount of Trp and Kyn through skin as a function of pH (*n* = 6). (**B**) Trp/Kyn ratio, corresponding to the data in (**A**). (**C**) Illustration of the dominating ionization species of Trp and Kyn at different pH values (numbers in the aromatic rings are in %). Values of p*K*_a_ of free fatty acids (FFA) in SC lipid model mixtures are from Ref.^47^.

The reason for investigating the Trp and Kyn permeability at different pH values mainly stems from the expectation that the pH of the aqueous microenvironment in the skin in vivo can vary. Healthy human skin has a quite broad pH range from pH of 4.5 to 6.0 depending on the body site, gender, age, and skin color^15–17^. In the case of skin barrier disruption, such as acute wound, the pH of the aqueous microenvironment can be significantly different with reported values as high as 9.0^18^. In particular, it is well known that cancers change the pH in their very local microenvironment. Further, for extraction purposes, extreme pH values is sometimes encountered; for example a pH value of 13 (0.1 M NaOH) has been used to sample isomers of urocanic acid from human skin in vivo in the past^30^. Taken together, it is clear that pH fluctuations, during in vivo topical skin sampling in clinical settings, is a very relevant aspect to consider. Therefore, we investigated the effect of pH on the Trp and Kyn permeability through skin membranes placed in media with controlled pH values ranging from 2.0 to 12.0. In these Franz cell experiments, the lower donor chamber was filled with buffer solution containing 1 mM Trp and Kyn, while the upper receptor chamber was filled with the same buffer without Trp and Kyn. Here, it should be pointed out that the experiments were designed to not have a gradient in the pH (i.e., the same pH value on both sides of the membrane) with the aim of obtaining stronger and clearer effects of the pH. After that, the Trp and Kyn were sampled from the receptor chamber over time to obtain the cumulative permeated amounts. The results from these experiments are presented in Fig. 2A, while Fig. 2B shows the corresponding Trp/Kyn ratio at different pH values, which were calculated from the cumulative values obtained for individual membranes and then averaged. Figure 2C illustrates the distribution of ionization species of Trp and Kyn at the investigated pH values (calculated by ChemAxon database and given in %). Note that in some cases in Fig. 2 (and Table 1), the number of calculated Trp/Kyn ratio is lower than the number of replicates (*n*) due to the fact that the Trp and Kyn concentrations were below the quantification limit. In these cases, the concentration was set to zero and the Trp/Kyn ratio lost its meaning and thus not considered for the calculation of the mean value of Trp/Kyn ratio. For clarity, the actual number of values used to calculate the Trp/Kyn ratio were: *n* = 1 (8 h) for pH 2.0; *n* = 1 (30 min, 1 h, 2 h), and *n* = 5 (4 h, 8 h) for pH 5.5; *n* = 1 (30 min, 1 h), *n* = 5 (2 h), and *n* = 6 (4 h, 8 h) for pH 7.4; *n* = 1 (2 h), *n* = 3 (4 h, 8 h) for pH 8.8; *n* = 2 (30 min, 1 h), *n* = 3 (2 h), and *n* = 4 (4 h, 8 h) for pH 12.0.

**Table 1:** Summary of the results from the permeability studies of Trp and Kyn (cf. Fig. 2). Note that the Trp/Kyn ratio is based on the cumulative amount of each permeant in the receptor solution after 8 h or 45 h.

pH	Analyte	Cum_8h_ (nmol cm^−2^)	Trp/Kyn (8 h)	Cum_45h_ (nmol cm^−2^)	Trp/Kyn (45 h)
2.0	Trp	0.11 (*n* = 6)	0.8 (*n* = 1)	0.40 ± 0.26 (*n* = 6)	0.1 ± 0.03 (*n* = 3)
	Kyn	0.66 ± 0.23 (*n* = 6)		6.84 ± 2.29 (*n* = 6)	
5.5	Trp	0.83 ± 0.28 (*n* = 6)	2.3 ± 0.5 (*n* = 5)	4.11 ± 1.19 (*n* = 3)	1.1 (*n* = 2)
	Kyn	0.35 ± 0.22 (*n* = 6)		3.21 ± 1.43 (*n* = 3)	
7.4	Trp	1.36 ± 0.21 (*n* = 6)	1.6 ± 0.2 (*n* = 6)	11.71 ± 1.39 (*n* = 3)	1.0 ± 0.1 (*n* = 3)
	Kyn	1.02 ± 0.25 (*n* = 6)		11.67 ± 1.74 (*n* = 3)	
8.8	Trp	0.87 ± 0.14 (*n* = 6)	1.7 ± 0.1 (*n* = 3)	10.68 ± 2.17 (*n* = 3)	1.2 ± 0.03 (*n* = 3)
	Kyn	0.28 ± 0.12 (*n* = 6)		9.22 ± 1.96 (*n* = 3)	
12.0	Trp	3.38 ± 0.85 (*n* = 6)	1.4 ± 0.1 (*n* = 4)	31.79 ± 9.69 (*n* = 6)	1.5 ± 0.2 (*n* = 6)
	Kyn	2.26 ± 0.89 (*n* = 6)		27.94 ± 9.90 (*n* = 6)	

A closer evaluation of the permeability data (Fig. 2A) shows that the cumulative amounts of either Trp or Kyn obtained after 8 h are similar at pH values of 5.5, 7.4, or 8.8 (i.e., no statistically significant difference). This conclusion is encouraging as it implies that the permeation behavior of Trp or Kyn will not change significantly if the pH of the skin surface sampling medium varies. In fact, in a clinical setting it is very likely that the pH will fluctuate depending on the type of disease, or due to other metabolic/biochemical challenges, or type of sampling methodology (occlusion or not, etc.). It should be noted, however, that the biological variation of the skin membranes can be relatively high. This variation may mask subtle pH effects on the ionization state of the permeants, which in principle should influence the skin permeability at pH values between 5.5 and 8.8. Based on the theoretical ionization profiles for Trp and Kyn (Fig. 2C), the majority of both permeants is expected to be zwitterionic at pH 5.5, 7.4 and 8.8. In addition, the pH can affect the charge status of the molecular components constituting the skin barrier. At pH levels of 5.5, 7.4 and 8.8, the skin is considered to be negatively charged^31^. Taken together, any effects on the skin permeability of Trp or Kyn, due to pH induced changes of either the ionization state of the permeants or the skin barrier molecular components, cannot be clearly observed at pH 5.5, 7.4, and 8.8 (Fig. 2A). In contrast, at pH 2.0, the cumulative amount of Trp is very low, while at pH 12.0, both Trp and Kyn show a very high permeability. In fact, at pH 12.0, the permeability of both Kyn and Trp was approximately 4 times higher as compared to all other pH values investigated. This significant increase of the skin permeability of both Trp and Kyn at pH 12 is probably due to disintegration of the skin barrier, for example, by alkaline hydrolysis of protein and lipid structures of SC, such as the cornified lipid envelope^32^.

The cumulative amount of permeated Trp is higher as compared to corresponding value of Kyn at all investigated pH values, except for pH 2.0. An obvious outcome of this finding is that the sampled Trp/Kyn ratio, presented in Fig. 2B, is higher than unity for all pH values, except for pH 2. However, the Trp/Kyn ratio changes over time; in particular over the first few hours and then levels out after approximately 4 h when a ratio close to two, or slightly below, is obtained. One likely reason for the initial fluctuations of the permeability data is that a stable hydration degree of the skin membrane is not reached immediately, which is required for steady state permeability values. Therefore, considering the observed initial fluctuating data on the Trp/Kyn ratio, an important conclusion is that these biomarkers should be sampled for at least 4–8 h in a clinical setting to reduce the variation of the Trp/Kyn ratio.

To further investigate the variability of the Trp/Kyn ratio over time, we performed prolonged permeability experiments over 45 h. The results from these experiments are summarized in Table 1 in terms of cumulative amounts and Trp/Kyn ratio, both after 8 h and 45 h (see Fig. S1 for plotted permeation profiles from these experiments).

Overall, Table 1 shows that the Trp/Kyn ratios, based on the cumulative amount of permeated Trp and Kyn after 8 h, varies between 0.8 and 2.3 depending on the pH value, while the average Trp/Kyn ratio after 8 h is equal to 1.7 ± 0.2 (*n* = 19). These values, obtained after 8 h, can be compared to the corresponding values obtained after 45 h, which varies between 0.1–1.5 with an average equal to 1.0 ± 0.1 (*n* = 17), see Table 1. In conclusion, the Trp/Kyn ratios approaches values close to unity at longer sampling times. Translating these in vitro result into the in vivo situation, the Trp/Kyn sampling ratio found on the surface of skin should be between 1–2 if the Trp and Kyn concentrations generated in the viable epidermis are similar. However, it is not likely that the concentrations of these biomarkers are the same in the real in vivo situation. For example, the reported Trp/Kyn ratio in healthy human blood is approximately equal to 30^33–36^, (see Table S1 for a detailed compilation). If we assume that a similar Trp/Kyn ratio is existing in the viable epidermis, based on the present results with a Trp/Kyn ratio between 1–2, it is expected that the Trp/Kyn ratio sampled from the skin surface should be approximately between 30–60. To the best of our knowledge, only one study has reported a Trp/Kyn ratio sampled from skin surface, which was found to be 338 in healthy people^37^. This relatively high Trp/Kyn ratio suggests that the amount of endogenous Trp inside the skin membrane, or on its surface, strongly influence the non-invasively sampled Trp/Kyn ratio. This is a very important aspect to consider, which is addressed in the following section.

Focusing on the data obtained at pH 2.0 (Fig. 2 and Table 1); at this low pH level, Trp was only detected in 1 out of 6 experiments after 8 h and in this case the cumulative amount was very low. On the other hand, the permeated cumulative amount of Kyn at pH 2.0 was similar as compared to the corresponding values from the other pH levels (Fig. 2A and Table 1). There are several possible reasons for these observations. Firstly, the dominating form of Trp, at pH 2.0, has a neutral carboxyl group and a positively charged ammonium group, while the zwitterionic form exists to a lower degree (Fig. 2C). The dominating form of Kyn at this pH has negatively charged carboxyl group (p*K*_a_ = 1.2 for the carboxylic acid of Kyn, which is very low) and two positively charged ammonium groups, giving the molecule a net positive charge. Effectively, this difference in ionization state between Trp and Kyn (Fig. 2C) results in a significant difference in their logD values, where Kyn (logD = − 3.1) is expected to be very hydrophilic while Trp (logD = − 1.5) is expected to be only moderately hydrophilic. In theory, this difference could result in that the majority of Trp or Kyn molecules permeates via different pathways across the skin barrier. However, in the case of zwitter and multi-charged ions, such as amino acids and their derivatives, it is not trivial to foresee what form results in the highest skin permeability^19^. For example, at pH 7.4 it was shown that net positively charged amino acids, such as arginine, had greater permeability, as compared to net negatively charged or zwitter ionic amino acids^19^. This finding can be explained by that the SC barrier, at physiological conditions (pH 7.4), is net negatively charged and permeation-selective towards cations^31^. However, at pH 2.0, the SC barrier is net positively charged and permeation-selective towards anions^31^, which is not the dominating form of neither Trp nor Kyn at pH 2.0.

In general, considering that the skin barrier is predominantly provided by the extracellular multilamellar lipids, it is reasonable to assume that passive diffusion for a particular ionizable molecule should be greater for the less charged form, as compared to its charged form, assuming that the less charged form is more lipophilic^8^. Thus, considering that Trp is relatively more lipophilic at pH 2.0, including the fact that Trp has a hydrophobic side group that may partition well into a lipid lamellae, it may be expected that Trp would permeate to a higher degree via free-volume and lateral diffusion through and along the lipid bilayers, respectively^23^. However, this reasoning is in strict disagreement with the observed results, showing very low skin permeability of Trp at pH 2.0. Hypothetically, Trp may be enriched inside the SC membrane by some specific interaction that is particularly enhanced at pH 2.0, such as enhanced partitioning in the lipid lamellar matrix or strong interaction with the cornified tissue, which then would act to minimize the amount of Trp permeating across to the other side. However, irrespective of the pH value, Trp is overall hydrophilic (i.e., negative logD values in all cases) and therefore expected to mainly diffuse through imperfections in the multilamellar lipid matrix surrounding the corneocytes in the SC (i.e., via the so-called pore pathway) and through shunt pathways across the SC^23^. Importantly, these pathways are expected to still be present at pH 2.0. In other words, the observed low permeability of Trp at pH 2 remains an unresolved issue.

### The influence of endogenous Trp on the Trp/Kyn ratio

**Figure 3:**
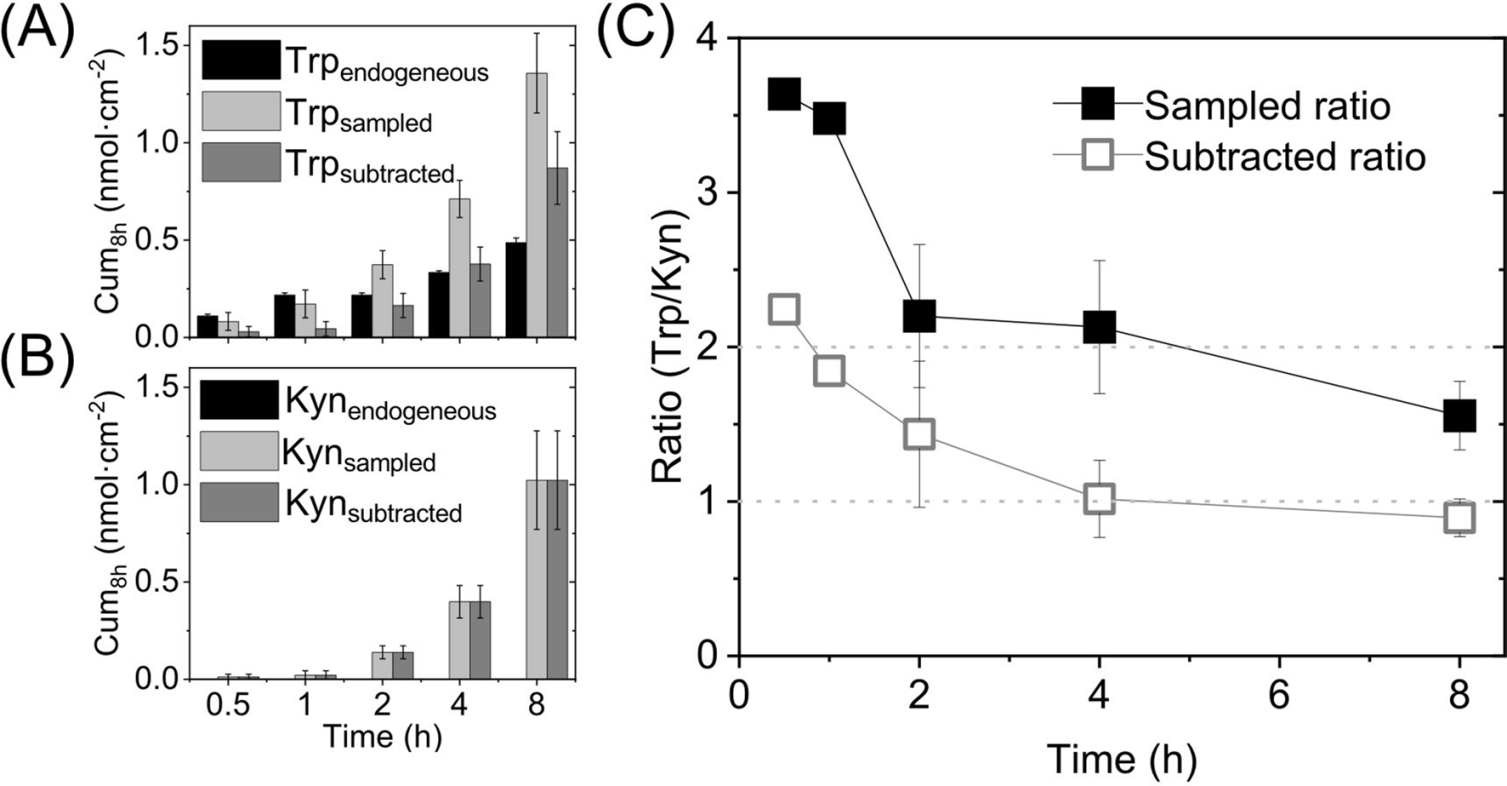
The influence of endogenous Trp on the Trp/Kyn ratio. Amounts of (**A**) Trp and (**B**) Kyn corresponding to the endogenous, sampled, and subtracted (= sampled − endogenous) quantities (*n* = 6, except for Trp_endogenous_ where *n* = 3). (**C**) Sampled and subtracted Trp/Kyn ratio based on the data in (**A**) and (**B**); *n* = 6 for the sampled ratio and *n* = 1 (30 min and 1 h); *n* = 3 (2 h); *n* = 6 (4 h); *n* = 6 (8 h) for the subtracted ratio.

It is known that the skin organ, specifically the outermost layer of the epidermis, generates its own small polar humectant molecules, commonly known as the natural moisturizing factor (NMF). The NMF compounds are very important for establishing a healthy skin barrier with normal hydration, softness, and pliability^26,29,38^. Trp is naturally present in the SC as a component of the NMF^19^. However, as concluded from the present results, endogenous Kyn was below the limit of detection (see Fig. 3B), which implies that Kyn should not be regarded as a component of the NMF. Considering these findings, we investigated the influence of endogenous Trp on the sampled Trp/Kyn ratio at pH 7.4 by performing 8 h experiments without addition of Trp and Kyn. The results from these experiments are presented in Fig. 3, which also includes results from experiments where Trp and Kyn were added to the lower donor chamber at concentrations of 1 mM for comparison. Note that the data denoted as subtracted in Fig. 3 represent the permeated (sampled) amount minus the endogenous amount.

As can be seen in Fig. 3A, a substantial amount of endogenous Trp is extracted from the skin membrane. In fact, even after 8 h of extraction time, the endogenous Trp fraction (0.49 ± 0.02 nmol cm^−2^, *n* = 3) constitute 40% ± 6% of the total amount that was sampled when 1 mM Trp was added to the lower Franz cell. On the other hand, the endogenous amount of Kyn was concluded to be below the limit of detection in all cases (see Fig. 3B). This finding indicates that establishing a limit of detection of Kyn in healthy human skin may require improved analytical procedures (beyond the scope of this study), which is important to consider in the case of comparing the Trp/Kyn ratio from healthy skin and cancer diseased skin in vivo. In any case, it is clear that the endogenous amount of Trp influences the sampled Trp/Kyn ratio (presented in Fig. 2B and Table 1). Therefore, it is reasonable to separate the sampled Trp/Kyn ratio, obtained under the influence of endogenous Trp, from the Trp/Kyn ratio obtained after subtracting the endogenous amount of Trp from the total sampled amount (referred to as the subtracted ratio in Fig. 3C). In conclusion, by adjusting for presence of endogenous Trp, the Trp/Kyn ratio is reduced and approaches unity. This finding is in line with the observation that the sampled Trp/Kyn ratio approaches unity after prolonged sampling times (see data for 45 h in Table 1) and can be rationalized by that complete extraction of endogenous Trp is achieved by extending the sampling time substantially (i.e., to 45 h). Importantly, the results from these in vitro experiments allow us to conclude that endogenous Trp will influence the sampled Trp/Kyn ratio in vivo, which is a valuable finding for any application of non-invasive monitoring and quantification of the Trp/Kyn ratio in a clinical setting.

### Skin membrane partitioning of Trp and Kyn as a function of pH (mass balance analysis)

A mass balance analysis was performed to better understand the Trp and Kyn skin permeability characteristics, including their partitioning behavior between the surrounding buffer medium and the interior of the skin membrane, at different pH values. In addition, a prerequisite for an unbiased evaluation of the Trp and Kyn skin permeabilities is that sink conditions are fulfilled during the Franz cell experiments and the validity of this assumption is thoroughly addressed by performing a mass balance analysis. For this, we determined (1) the amount of Trp and Kyn present in the lower donor chamber, (2) the amount permeated through the skin to the upper receptor chamber, and (3) the amount accumulated inside the skin membrane after 45 h permeability experiments. Here, it should be noted that these experiments were performed with addition of 15 mM NaN_3_ to avoid microbial degradation, in particular in the cases of pH 5.5, 7.4 and 8.8 (microbial degradation is not an issue at pH 2.0 and 12.0).

**Figure 4:**
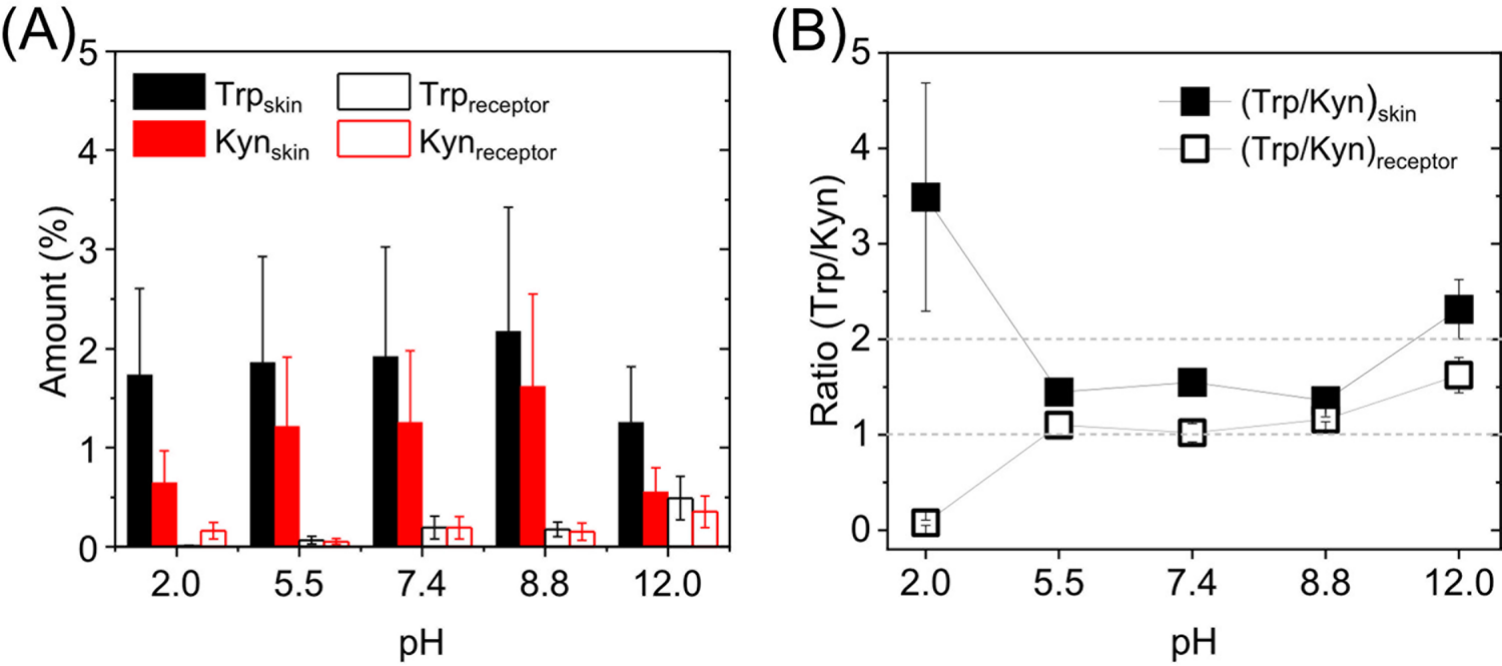
(**A**) Mass balance of Trp and Kyn between the skin membrane and the receptor solution as a function of pH (%). The number of replicates were *n* = 4 at pH 2.0; *n* = 3 at pH 5.5; *n* = 3 at pH 7.4; *n* = 3 at pH 8.8; *n* = 5 at pH 12.0. (**B**) The Trp/Kyn ratio based on the data in (**A**); *n* = 4 at pH 2.0; *n* = 3 at pH 5.5; *n* = 3 at pH 7.4; *n* = 3 at pH 8.8; *n* = 5 at pH 12.0 for (Trp/Kyn)_skin_ and *n* = 3 at pH 2; *n* = 3 at pH 5.5; *n* = 3 at pH 7.4; *n* = 3 at pH 8.8; *n* = 5 at pH 12.0 for the (Trp/Kyn)_receptor_.

Starting with the Trp and Kyn concentrations in the lower Franz cell chamber; irrespective of pH (pH of 2.0–12.0), the concentrations were concluded to be unaffected and close to constant during the permeability experiments. In fact, no statistically significant difference (*p* = 0.985) between the initial and final concentrations of Trp was found before and after the permeability experiments, see Table S2. However, at pH 12, a significant reduction of the concentration of Kyn was observed (*p* = 0.00046) where C_before_ = 0.95 mM ± 0.12 mM (*n* = 3) and C_after_ = 0.48 mM ± 0.05 mM (*n* = 6), see Table S2*.* Based on this finding, we performed systematic stability tests of Trp and Kyn at all relevant experimental conditions (32 °C and 45 h, see Table S3). From these control experiments, it is possible to conclude that Kyn, but not Trp, is susceptible for chemical degradation during exposure for prolonged times at pH 12 and 32 °C, while both Trp and Kyn are stable at all other pH values under the present experimental conditions. Still, even though Kyn is degraded over time at pH 12, it is safe to assume that the concentration of Kyn in the lower Franz cell remains sufficiently constant to maintain sink conditions. This is supported by the permeation profiles obtained at pH 12 (see Fig. S1), showing no indication of reduced flux as a result of depletion of the donor concentration due to degradation of Kyn. Thus, the first conclusion from this analysis is that the donor concentration of Trp and Kyn (i.e., 1 mM), can be assumed to be constant and not affected by depletion, which is one of two general requirements to maintain sink conditions. The second requirement is fulfilled if the concentration of Trp and Kyn, in the upper receptor solution, do not exceed 10% of their solubility limits (as a rule of thumb), which correspond to approximately 0.5–5 mM for Trp and 2–90 mM for Kyn (depending on the pH). In the present study, also this requirement was fulfilled with great margin. In fact, the concentrations of the permeants in the upper receptor chamber never exceeded 0.1 mM for any of the permeants and were generally significantly lower than this concentration. Finally, to complete the mass balance analysis, we determined the amount of Trp and Kyn inside the skin membranes after 45 h of permeability experiments. For clarity, the results are presented in Fig. 4 where the percentage of the accumulated amount of Trp and Kyn in the skin membrane and the receptor solution is presented with respect to the amount added to the donor solution (i.e., 6 mL of 1 mM = 6 µmol = 100%). Note that the amount of Trp and Kyn in the lower donor chamber is omitted in Fig. 4 as this parameter can be assumed to be constant.

Interestingly, from the mass balance analysis presented in Fig. 4, it is clear that the extracted amount of Trp is considerably higher as compared to the amount of extracted Kyn, irrespective of pH value. The amount of Trp accumulated inside the skin membrane is 1.73% ± 0.16% (*n* = 18) of the total amount, while the corresponding percentage for Kyn is 0.99% ± 0.14% (*n* = 18). In other words, based on experimental results, the accumulation of Trp in the skin membrane is higher as compared to Kyn. This observation can be rationalized based on the fact that Trp is nearly twice as hydrophobic as compared to Kyn, based on their Log D values (see Table S4), irrespective of the pH value. Indeed, both permeants are hydrophilic, but in relative terms, Trp is less so. Another reason for the observed higher amounts of Trp inside the skin membrane, as well as in the receptor solution, may be related to the fact that Trp is found endogenously in the skin membrane as part of the NMF. However, as shown in Fig. 3, the endogenous source of Trp is limited and significantly reduced over time and therefore expected to be insignificant in these mass balance experiments, which rely on extraction of Trp from skin membranes after a prolonged experimental time of 45 h. This is further supported by comparing the Trp/Kyn ratios after 8 h (close to 2) and 45 h (close to 1) in Table 1. Finally, it should be noted that the extraction of Trp and Kyn was performed by soaking the skin membranes for a limited time period (1 h) in an aqueous solution with pH 12 to limit any degradation of Kyn, which can occur for prolonged exposure times (45 h, see Table S3). Supposedly, this caveat might influence the results. However, since the Trp/Kyn ratios (Fig. 4B), corresponding to the skin membrane and the receptor solution, show no significant difference, it is not likely that Kyn degradation during the extraction procedure influenced the results to any significant degree.

Next, from Fig. 4A it can be concluded that the fraction of Trp or Kyn that permeates across the membrane to the receptor solution, is much lower as compared to the corresponding amount inside the skin membrane. More specifically, the amount of Kyn permeated is 0.20% ± 0.05% (*n* = 18), which is 5 times lower as compared to the amount found inside the skin membrane. The corresponding number for Trp is 0.21% ± 0.07% (*n* = 18), which is 8 times lower than the amount residing in the skin membrane. In other words, the permeated amount of Trp and Kyn is very similar after 45 h, while the accumulated amount of Trp inside the skin membrane is higher as compared to Kyn.

Finally, based on the results in Fig. 4A, the Trp/Kyn ratios, corresponding to the amounts extracted from the skin membrane and the amounts determined in the receptor solution, were calculated and presented in Fig. 4B. Due to the relatively high variations of the data, it is not possible to distinguish any significant differences between the ratios obtained for the various pH values, with the exception of the ratios obtained at pH 2. In the latter case, the ratio corresponding to the amounts extracted from the skin membrane is considerably higher than unity (3.5 ± 1.2; *n* = 4), while the receptor ratio is much lower than unity (0.078 ± 0.03; *n* = 3). Here, it should be noted that Trp was not detected in the acidic receptor solution at pH 2 in most cases, resulting in a very low (Trp/Kyn)_receptor_ ratio. Potentially, this could be due to acidic hydrolysis of Trp in the receptor solution, but this is not supported by the stability tests performed at pH 2, showing no reduction of the Trp concentration (see Table S3). Another possible explanation may be due to reduced skin permeability at lower pH values, for both permeants in general, but for Trp in particular (this is most clearly shown in Fig. S1). Possibly, a reduced skin permeability may be related to an increased partitioning and accumulation inside the skin membrane, in particular of the relatively more hydrophobic Trp molecule. However, the very high (Trp/Kyn)_skin_ ratio at pH 2, seems to be more related to a relatively low accumulation of Kyn inside the skin membrane observed in Fig. 4A.

### Influence of microbial degradation of Trp and Kyn on the sampling Trp/Kyn ratio

**Figure 5:**
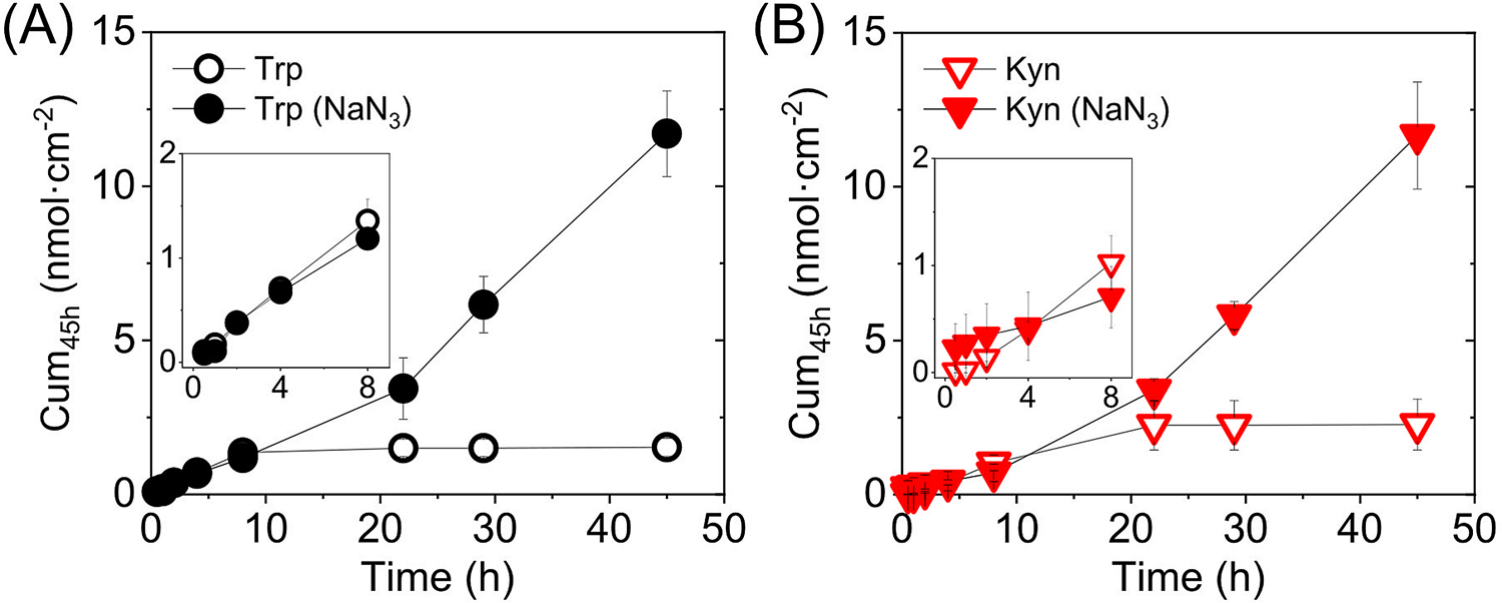
Cumulative permeated amount of (**A**) Trp and (**B**) Kyn through skin with (*n* = 6) and without (*n* = 3) presence of the antimicrobial agent sodium azide (15 mM NaN_3_).

In principle, several factors may affect the outcome of non-invasive topical sampling of Trp/Kyn ratio as a potential biomarker for skin cancer. Here, we would like to emphasize the important role of the skin microbiota with respect to the fate of topical biomarkers or transdermal permeants. This topic is rarely specifically considered when performing in vitro permeation studies. For example, according to the OECD guidelines for skin absorption in vitro, the permeation studies should not exceed 24 h due to possible skin disintegration. However, there is nothing mentioned regarding experimental procedures to handle the presence of microbiota and its potential influence on the permeation results^39^. To address this issue, with respect to microbial degradation of Trp and Kyn, we performed permeability experiments over 45 h without or with the established antimicrobial agent sodium azide (15 mM NaN_3_) added to both the donor and the receptor chambers^40^. The results from these experiments are presented in Fig. 5 for pH 7.4 and in Fig. S1 and S2 for all pH levels investigated (in all cases, the Trp and Kyn concentrations in the lower Franz cells chamber were 1 mM). Strikingly, in the case where NaN_3_ is not present, the curves of the cumulative permeated amount of Trp and Kyn levels out after certain time points at pH 7.4; i.e., after 8 h and 22 h, respectively. As clearly illustrated in Fig. 5, continuous permeation profiles of both permeants are obtained in the presence of the antimicrobial agent. In addition, similar results were obtained for buffers with pH 5.5 and 8.8 (Fig. S2), while no indication of microbial degradation of Trp and Kyn was observed in the case of the harsh pH values of 2.0 and 12.0 (Fig. S1). In other words, monotonically increasing permeation profiles over the entire 45 h time span were obtained without azide for both Trp and Kyn at pH 2.0 and 12.0 (Fig. S1).

Based on these results (Fig. 5, Fig. S1, and S2), it is possible to conclude that the skin microbiota degrades both Trp and Kyn at biologically relevant pH values, while addition of the microbial inhibitor NaN_3_, or highly acidic or alkaline pH levels, diminishes this process. Indeed, it is known that skin microbiota can metabolize and degrade Trp^37,41^. However, to our knowledge, this effect has not been shown for Kyn before. Further, the results show that the microbial degradation process occurs at moderately acidic (pH 5.5) and alkaline (pH 8.8) conditions, while being avoided at harsh conditions (pH 2.0 and 12.0). These are important findings that should be considered in the case of in vivo topical sampling of Trp and Kyn or other similar biomarkers. Interestingly, also the amounts of Trp and Kyn that was extracted from the skin membrane after 45 h of permeability experiments (at pH 7.4) were slightly lower without NaN_3_, as compared to the results obtained with NaN_3_. However, these differences were not statistically significant. For Trp, the extracted amounts were 69.6 ± 17.9 and 115.0 ± 19.5 nmol cm^−2^ (*p* = *0.161*), without and with NaN_3_, respectively. For Kyn, the corresponding values were 65.9 ± 15.5 and 75.3 nmol cm^−2^ ± 14.7 (*p* = 0.683), respectively.

For the purpose of this work, it is important to point out that the difference between the cumulative permeated amounts after 8 h of Trp or Kyn, with and without NaN_3_, is statistically similar (*p*-values for pH 5.5: *p* = 0.887 (Trp), *p* = 0.227 (Kyn), pH 7.4: *p* = 0.592 (Trp), *p* = 0.471 (Kyn), pH 8.8: *p* = 0.075 (Trp), *p* = 0.521 (Kyn), see Fig. S2). Further, the Trp/Kyn ratio, calculated from the cumulative permeated amount after 8 h (Fig. 5), with and without NaN_3_ at pH 7.4, was concluded to be statistically similar: 2.4 ± 0.9 with NaN_3_ (*n* = 3) and 1.6 ± 0.2 without NaN_3_ (*n* = 6).

Overall, the results from the permeation experiments, performed with and without NaN_3_, show that microbiota present on the skin surface, and potentially to some extent present inside the skin membrane, can strongly affect the Trp and Kyn amount sampled from the surface of the skin. In addition, the results indicate that the degradation rate of Trp and Kyn, due to microorganisms, dominates over the skin permeation rates after prolonged experimental times; i.e., 8 h or longer. Based on this, in order to overcome microbial degradation in vitro and in vivo at occluded conditions, the Trp and Kyn sampling time should not exceed 8 h and/or the sampling protocol should include an antimicrobial agent (which may be problematic for toxicity reasons).

### Comparison of experimental and theoretical Trp and Kyn skin permeability coefficients

**Figure 6:**
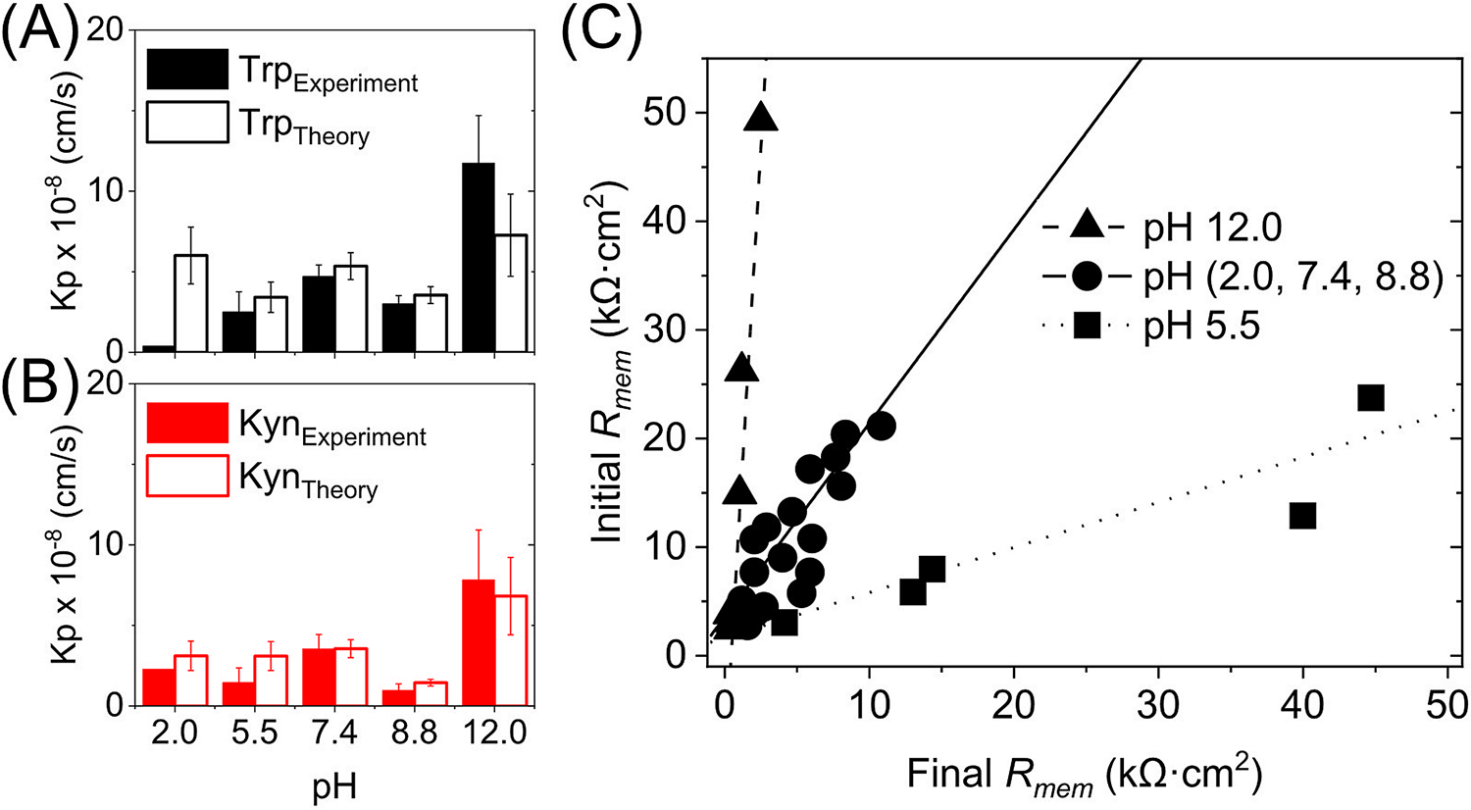
Experimental (*n* = 6, except for pH 5.5 where *n* = 5) and theoretical permeability constants $K_{p}$ for (**A**) Trp and (**B**) Kyn as a function of pH. (**C**) Correlation between initial (0 h) and final (after 45 h) skin membrane resistance ($R_{mem}$) for various pH values; *n* = 6 at pH 12, *n* = 18 at pH 2.0, 7.4, 8.8, *n* = 5 at pH 5.5.

From the experimental permeation data obtained after 8 h, the Trp and Kyn permeability coefficients ($K_{p}$) were calculated according to Eq. (1) and compared with the corresponding values obtained from theoretical calculations based to the four permeation pathways theory^23^ (see Table 2 and Fig. 6A, B). The theory assumes the following four permeation pathways: free-volume and lateral diffusion of lipophilic molecules inside the extracellular SC lipid lamellar matrix, transport of relatively large (and small) hydrophilic permeants through shunts (represented by both hair follicles and sweat ducts), and finally diffusion through nanosized defects of the SC lipid lamellar matrix, inside which aqueous medium can assist passage of small hydrophilic permeants^23^. All theoretical calculations follow previously published work^23,42–46^, and are detailed in the Supplementary Information. In brief, the permeability constants for Trp and Kyn can be calculated based on known, or estimated, physiochemical variables, such as molecular weight and size, distribution coefficients at different pH values (i.e., log D values), and diffusion coefficients in the different SC compartments. These input parameters are used together with estimated intrinsic properties of the skin barrier, such as the SC thickness, area fraction covered by shunts (i.e., hair follicles and sweat ducts), and imperfections of the continuous lipid lamellar matrix. Notably, the latter property is modelled as dynamic pores that are hydrophilic in nature and can be characterized in terms of porosity and tortuosity, which can be related to the skin membrane resistance, $R_{mem}$. Therefore, the $R_{mem}$ was determined for all skin membranes employed in the present study, before (initial $R_{mem}$, 0 h) and after (final $R_{mem}$, 45 h) the permeability experiment (see Fig. 6C). Here, it should be pointed out that the calculations, performed with $K_{p}$ values obtained after 8 h together with either the initial or the final $R_{mem}$ values, resulted in similar fallouts in general. However, in the present evaluation, only the results based on the initial $R_{mem}$ values are considered due to the fact that they were most consistent with previously reported data from similar evaluations in terms of the pore radius $r$ (estimated between approximately 10–40 Å), the ratio between the hydrodynamic radius of the permeant and the pore radius $\lambda_{p}$ ($\lambda_{p} <$ 0.4), and the hindrance factor $H(\lambda_{p} )$ (estimated between 0.13–62)^23,43–46^.

**Table 2:** Experimental and theoretical Trp and Kyn permeability constants ($K_{p}$). The theoretical calculations were obtained according to the four permeation pathways theory (see Supplementary Information); *n* = 6, except for pH 5.5 where *n* = 5).

Conditions	$K_{p} \times$ 10^–8^ (cm/s)	
	Trp	Kyn
**pH 2.0**		
Experiment	0.39	2.29 ± 0.79
Theory	6.01 ± 1.76	3.11 ± 0.91
**pH 5.5**		
Experiment	2.52 ± 1.23	1.46 ± 0.91
Theory	3.42 ± 0.97	3.10 ± 0.94
**pH 7.4**		
Experiment	4.71 ± 0.71	3.55 ± 0.88
Theory	5.35 ± 0.83	3.56 ± 0.56
**pH 8.8**		
Experiment	3.02 ± 0.49	0.97 ± 0.40
Theory	3.55 ± 0.52	1.45 ± 0.21
**pH 12.0**		
Experiment	11.75 ± 7.83	7.83 ± 3.09
Theory	7.27 ± 2.55	6.82 ± 2.40

In general, there is good agreement between the experimental permeability constant ($K_{p}$) and its theoretically calculated counterpart (Fig. 6A,B). The experimental $K_{p}$ values are, however, systematically lower as compared to the calculated values, in particular at the more acidic pH levels. One exception to this is the results corresponding to pH 12.0, where the opposite is true. The latter finding is likely due to alkaline hydrolysis and disintegration of the SC barrier at this harsh pH level, resulting in increased permeability and a drastic decrease of the skin membrane resistance ($R_{mem}$), see Fig. 6C. This is, however, not fully reflected by the theoretical model.

The (unknown) reason for the very low Trp permeability at pH 2.0 was discussed above in relation to Fig. 2 and Table 1. In brief, at this acidic pH, the reduced Trp skin permeability is neither reflected by the change of $R_{mem}$, which is similar for the cases of pH 2.0, 7.4, and 8.8 (see Fig. 6C), nor the theoretical model that predicts a relatively high value of $K_{p}$ at pH 2.0 (Fig. 6A).

At pH 5.5, the theory predicts relatively low values of $K_{p}$, both for Trp and Kyn, as compared to any other pH levels (except for pH 8.8 for Kyn). This finding is in line with the observed change of $R_{mem}$ at pH 5.5, which distinctively increased during the permeability experiment (see Fig. 6C). This implies that the integrity of the skin barrier is maintained, or even increases, at pH 5.5 in terms of its capacity to oppose transport of both hydrophilic permeants and ions.

Taken together, the experimental and theoretical permeability constants are on the same order of magnitude and in good agreement with each other. This conclusion is encouraging as it opens up for theoretical predictions of permeability coefficients of also other small hydrophilic biomarkers that may be of interest, both for skin cancer and other skin diseases. In addition, the experimentally determined Trp permeability constants fall in the range 0.4–12 × 10^–8^ cm/s, depending on the pH (see Table 2). This is in relatively good agreement with previously published data obtained with hairless mouse skin in vitro, which were reported to be 0.4 × 10^–8^ cm/s at physiological pH^19^. However, at pH 7.4, the present Trp permeability coefficient is significantly higher (equal to 4.7 × 10^–8^) as compared to the value obtained with mouse skin. This is probably due to biological variation between species (i.e., pig skin versus mouse skin). Also, mouse and pig skin may have different levels of endogenous Trp naturally present in their skin, which could influence the determined permeability coefficient if not adequately considered, as shown in this work. On a more general note, the present permeability results, both for Trp and Kyn, are in a good agreement with previously reported permeability constants for other hydrophilic permeants with similar molecular weights. For example, reported values for mannitol (MW = 182 g/mol, Log K_o/w_ = -3.1) are 0.7 × 10^–8^ (pig skin) and 1.4 × 10^–8^ (human skin), while the corresponding value for diethylcarbamazine (MW = 199 g/mol, Log K_o/w_ = − 2.0) is reported to be 13 × 10^–8^ (human skin)^23^.

**Figure 7:**
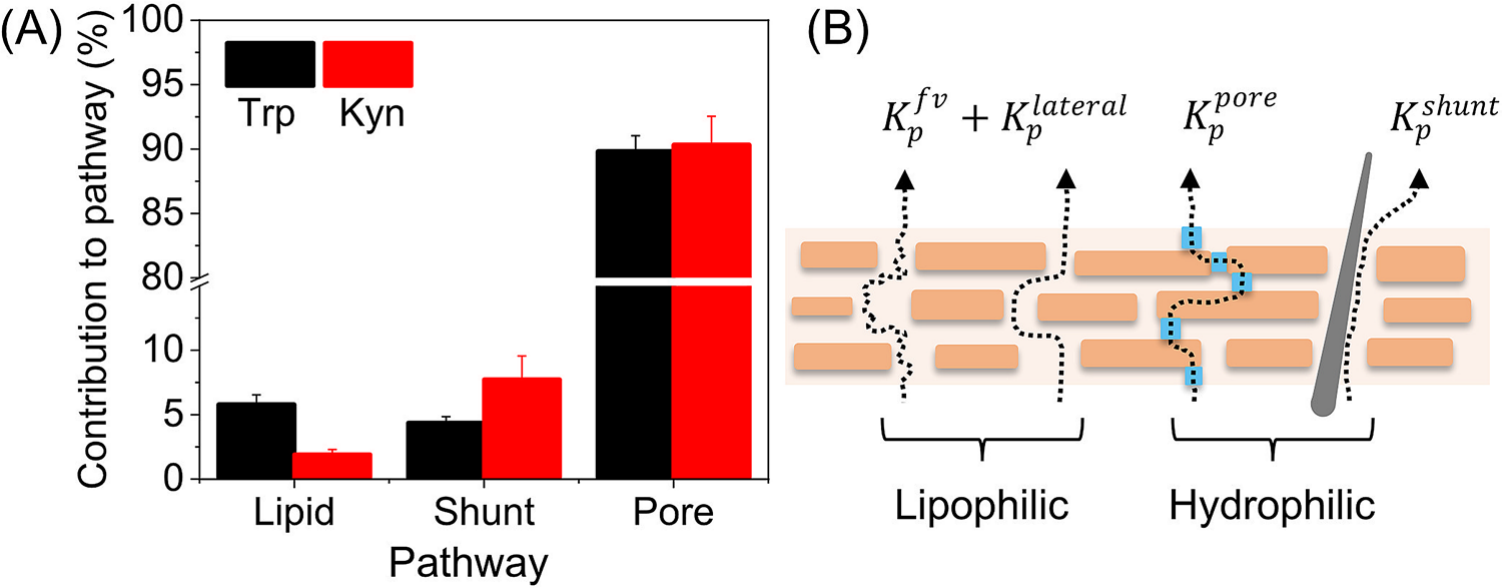
(**A**) Theoretical contribution of each permeation pathway of the total skin permeability of Trp and Kyn (%) based on data from pH 2.0, 5.5, 7.4, 8.8, and 12.0, see Table S7. The lipid category represents both the free-volume and the lateral diffusion pathways via the lipid lamellar matrix. (**B**) Schematic illustration of the different diffusion pathways considered in the four permeation pathways model.

The fact that we observe good agreement between the permeability constants from experiment and theory allows for a closer analysis of the contribution from the different diffusion pathways according to the theoretical four permeation pathways model. The general outcome of this analysis is presented in Fig. 7A, while a detailed compilation of the calculated permeability constants for both Trp and Kyn from each pathway (i.e., $K_{p}^{fv}$, $K_{p}^{lateral}$, $K_{p}^{shunt}$, and $K_{p}^{pore}$) and their contribution to the total permeability constant ($K_{p}$) is presented in Table S7.

The most prominent outcome from this analysis (Fig. 7A) is that, according to four permeation pathways theory, about 85–94% of Trp and 82–93% of Kyn permeate via the pore pathway (including all pH levels, see Table S7). This pathway should be envisaged as a collection of dynamic defective regions (imperfections) in the multilamellar lipid matrix in the SC with size dimensions in the range of roughly 1–10 nm. Structural defects in this continuous lipid matrix, such as grain boundaries and lipid vacancies leading to so-called fault dislocations, etc., are commonly observed in lamellar lipid systems that are exposed to high local membrane curvature, lateral phase separation, or osmotic stress^23^. Speculatively, these imperfections allow for local pore openings where aqueous medium can act to support transport of hydrophilic molecules, such as Trp and Kyn, and ions^23,43–46^.

Another conclusion from Fig. 7A is that the shunt pathway also contributes significantly to the overall permeability of both Trp (3–6%) and Kyn (6–14%); see Table S7. In particular for Kyn, which is slightly more hydrophilic as compared to Trp. Thus, the results imply that the passage through the shunt pathway should not be neglected or underestimated for Trp and Kyn. On the other hand, the relatively more hydrophobic Trp molecule is expected to permeate to a higher degree via the free-volume and lateral diffusion pathways in the lipid lamellar matrix of SC (Fig. 7A and Table S7). In contrast to the pore pathway, free-volume diffusion via the lipid domains is hypothesized to occur through density fluctuations in the lipid chains, which open up about 1 nm small hydrophobic voids, allowing for diffusion of relatively small lipophilic permeants^23^. In principle, the hydrophobic indole structure of the Trp molecule is likely to explain the relatively higher degree of diffusion via the lipid pathway for this molecule as compared to Kyn, which structure does not entail an indole moiety but rather a more hydrophilic residual structure as a consequence indole ring opening.

The theoretical analysis of the contribution of each permeation pathway (Fig. 7A) warrants for a reassessment of the observed, relatively low, skin permeability at acidic pH values (i.e., 2.0 and 5.5, see Table 1), including the distinctive increase of $R_{mem}$ at pH 5.5 (Fig. 6C). A relevant starting point for this is to conclude that diffusion via pores (i.e., lipid imperfections) represents the main diffusion pathway, both for Trp and Kyn (Fig. 7A). Further, this pathway is also available for diffusion of ions (i.e., electrical charge carriers) and therefore expected to be the main contributor for passage of electrical currents. Based on this, it can be hypothesized that the observations of low skin permeability at pH 2.0 and 5.5, and the increase of $R_{mem}$ at pH 5.5, are most likely related to pH induced alterations of the lipid matrix of the SC barrier. Tentatively, these alterations lead to a reduction of lipid imperfections rather than, for example, alterations of the physicochemical properties associated with the shunt pathway. Reported apparent p*K*_a_ values of free fatty acids in model mixtures of SC lipids are suggested to be between 6.2–7.3 based on FTIR measurements of the spectral band intensities of the protonated and deprotonated carboxylic acid moiety^47^. On this basis, at pH 2.0 and 5.5 the majority of the free fatty acids are protonated with reduced coulombic repulsive interactions between their headgroups, which favors a more close packed and ordered SC lipid lamellar structure. This mechanism, if correct, would most likely minimize lipid imperfections and thereby reduce the Trp and Kyn skin permeability, which is in line with the observed results at pH 2.0 and 5.5. However, the discrepancy between the changes of $R_{mem}$, which decreases at pH 2.0 and increases at 5.5 (Fig. 6C), is more complicated to explain. A possible explanation of the non-existing increase of $R_{mem}$ at pH 2.0 (Fig. 6C), is that acidic hydrolysis, for example, of amide linkages of ceramides or disintegration of protein structures of SC, may occur at pH 2.0. This would counteract the effect of increased electrical resistance due to protonated and close packed free fatty acids of the SC lipid matrix. Speculatively, this would lead to an overall reduction of the electrical resistance.

## Concluding remarks

The cancer biomarker IDO1 has been reported to increase the conversion of Trp to Kyn^10–12^, which implies that non-invasive sampling of the Trp/Kyn ratio from the skin surface is a promising skin cancer biomarker. To investigate the possibility of utilizing this concept, we have investigated the general skin permeability characteristics of Trp and Kyn by performing Franz cell diffusion studies with 1 mM Trp and Kyn added to the lower chamber and sampling from the upper chamber. The most important conclusions are summarized below.

- From permeability experiments performed at pH 2.0, 5.5, 7.4, 8.8, and 12.0 it is possible to conclude that Trp and Kyn follow a similar transdermal transport behavior, irrespective of pH value (Fig. 2A). Even though the two permeants diffuse at approximately similar rates, the Trp amount is in general higher as compared to Kyn due to endogenous Trp, which ultimately leads to Trp/Kyn ratios slightly higher than unity (Fig. 2B). One exception of these general conclusions is that the Kyn permeability at pH 2.0 is higher as compared to the Trp permeability for a reason that remains unclear. Knowing that pH of human skin varies, a relatively stable Trp and Kyn permeation over the pH range 5.5–8.8, is a positive result for potential Trp/Kyn sampling in clinical setting.
- The skin permeability of Trp and Kyn is in general lower at reduced pH levels (i.e., 2.0 and 5.5) as compared to neutral or slightly alkaline (i.e., pH 7.4 and 8.8), while a pH value of 12.0 results in significant increase of transdermal transport of both Trp and Kyn due to disintegration of the skin barrier.
- The high variability of the initially sampled Trp/Kyn ratio is due to endogenous Trp, while endogenous Kyn was never detected (Fig. 3). This finding explains why the Trp/Kyn ratio approaches unity after prolonged sampling times during which the pool of endogenous Trp is exhausted and therefore contributes to a lower extent to the sampled Trp/Kyn ratio (Fig. 3 and Table 1). Importantly, endogenous Trp needs to be considered in any application of non-invasive monitoring and quantification of the Trp/Kyn ratio in a clinical setting.
- From a mass balance evaluation at various pH levels, it is clear that Trp, in general, both accumulates inside the skin membrane and permeates across it to a higher degree as compared to Kyn (Fig. 4A). Further, the amounts accumulated inside the skin membrane are always higher as compared to the permeated amounts, both for Trp and Kyn (Fig. 4A). However, the Trp/Kyn ratio is similar in all cases except for pH 2.0, irrespective of being based on the amount accumulated inside the skin membrane or permeated across the membrane (Fig. 4B).
- The degradation of Trp and Kyn due to microbiota residing inside or on the skin surface needs to be considered in any application of non-invasive monitoring and quantification of the Trp/Kyn ratio in a clinical setting (Fig. 5, Figs. S1, and S2). The present in vitro experiments illustrate that Trp/Kyn ratio is not altered by microbiota during the initial 8 h. Therefore, the sampling protocol in vivo should be relatively short or include an antimicrobial agent.
- Both for Trp and Kyn, the experimental and theoretical permeability constants are in good agreement with each other at various pH values. This promising conclusion opens up for theoretical predictions of permeability coefficients of other small hydrophilic biomarkers that may be of interest, both for skin cancer and other skin diseases.
- Trp and Kyn are hydrophilic molecules, which according to four permeation pathways theory mainly permeate through porous lipid defects (roughly 82–94%). However, the more hydrophobic indole ring of Trp is suggested to result in a small but noticeable relative increase of Trp transport via free-volume and lateral diffusion pathways. On the other hand, the shunt diffusion pathway is proposed to be relatively more favorable for the more hydrophilic Kyn molecule.

## Supplementary Information

Supplementary Information.
