# Supplementary material for: Non-invasive skin sampling of tryptophan/kynurenine ratio in vitro towards a skin cancer biomarker
Source: Sci Rep. 2021 Jan 12;11:678. doi: 10.1038/s41598-020-79903-w (PMC7803776; doi:10.1038/s41598-020-79903-w)
Supplement: Supplementary file 1 — Supplementary Information. [file 41598_2020_79903_MOESM1_ESM.pdf]

Supplementary Information

Non-invasive skin sampling of tryptophan/kynurenine ratio *in vitro* towards a skin cancer biomarker

Skaidre Jankovskaja[1,2], Johan Engblom[1,2], Melinda Rezeli[3], György Marko-Varga[3], Tautgirdas Ruzgas[1,2] and Sebastian Björklund[1,2,*]

[1]Department of Biomedical Science, Faculty of Health and Society, Malmö University, 205 06 Malmö, Sweden

[2]Biofilms - Research Center for Biointerfaces, Malmö University, 205 06 Malmö, Sweden

3Clinical Protein Science and Imaging, Department of Biomedical Engineering, Lund University, Lund, Sweden

*Corresponding author: sebastian.bjorklund@mau.se

Table S1. Summary of tryptophan (Trp) and kynurenine (Kyn) quantities reported in the literature in human and mice blood, skin surface and the site of tumors.

Condition	Sample origin	*n*	Trp (µM±SD)	Kyn (µM±SD)	Trp/Kyn	Ref.
Healthy	Blood	49	73.0±14.9	1.9±0.6	38	1
Healthy	Blood	17	68.6±25.0	2.3±0.7	30	2
Healthy	Blood	39	56.3±38.2	3.0±0.9	19	2
Healthy	Blood	135	58.0±9.6	1.6±0.3	36	3
Healthy	Blood	24	52.9±11.0	2.0±0.4	26	4
Healthy	Skin surface	19	0.98	0.0029	338	5
Diseased	Blood (melanoma)	87	57.0±10.7	2.6±1.0	22	1
Diseased	Blood (renal cell carcinoma)	91	44.4±14.4	6.6±2.1	7	6
Diseased	Blood (breast Cancer)	33	48.9±11.0	2.3±0.2	21	4
Diseased	Blood (melanoma); mice	36	94.3	0.8	112	7
Diseased	Tumor (melanoma); mice	5	109.36	3.8	29	7

Table S2. Comparison of tryptophan (Trp) and kynurenine (Kyn) concentrations in the lower Franz cell chamber, before and after the permeation experiment. The results are represented as a mean value ± standard error of the mean (C_{before} n=3 and C_{after} n=6). C_{before} refers to the concentration of the aliquot taken before starting Franz cell experiment (kept in the fridge at +4 °C until the day of analysis (~60 h). C_{after} is the concentration of the aliquot taken from the lower Franz cell chamber after 45 h of the permeation experiment (32 °C) and storing it in the fridge (+4 °C) overnight prior the analysis. C_{before} and C_{after} were estimated the same day of analysis.

pH	Tryptophan			Kynurenine		
	C_{before} (µM)	C_{after} (µM)	C_{before} vs C_{after}	C_{before} (µM)	C_{after} (µM)	C_{before} vs C_{after}
2.0	854.0±20.0	1021.3±66.0	p=0.58499	910.6±24.6	996.5±58.1	p=0.99482
5.5	1133.2±32.6	1133.9±09.0	p=1	1113.6±30.4	926.2±30.4	p=0.60462
7.4	1010.9±05.7	1082.9±29.9	p=0.99636	1294.4±41.5	1081.1±72.5	p=0.42873
8.8	1155.0±05.0	1089.7±55.1	p=0.99825	1190.4±06.2	984.9±70.9	p=0.47974
12.0	836.3±119.4	940.7±47.8	p=0.95457	953.6±122.6	476.5±47.5	p=0.00046
Range: 2.0-12.0	997.9±41.7	1022.24±39.9	p=0.98456	1092.5±44.7	893.0±46.5	p=0.02356
Range: 2.0-8.8	1038.3±37.0	1042.6±47.9	p=0.99991	1127.3±44.1	997.2±30.4	p=0.08371

Table S3. Stability of tryptophan (Trp) and kynurenine (Kyn) at different pH values used in the permeability studies. The stability of Trp and Kyn was evaluated by placing the analytes in vials filled with 475 µL of PBS or NaCl solution adjusted to a particular pH. NaN_3 (15 mM) was added to the solutions at pH values of 5.5, 7.4, and 8.8. After that, 25 µL of 0.2 mM stock of Trp or Kyn was pipetted into vial resulting into a total volume of 500 µL with concentrations of 10 µM of Trp and Kyn. Next, the vials were placed in a water bath (32 °C) and kept there for 45 h. Two control vials (one with 10 µM of Trp and another one with 10uM of Kyn) were prepared in 20% MeOH simultaneously, and stored at -20 °C for 45 h. After 45 h, the concentration of Trp and Kyn was determined by HPLC and the change Δ (%) was calculated from the following expression: $\Delta(\%) = C_1[\mu M]/C_2[\mu M] \times 100\%$, where C_1 is the concentration of the analyte after exposing it to a particular pH for 45 h (at 32 °C) and C_2 is the concentration of the analytes estimated in the sample prepared in 20% MeOH and kept for 45h in the freezer at -20 °C (control sample, C_2=10.2 for Trp and C_2=10.9 for Kyn).

Condition	Concentration (µM)		Δ (%)	
	Trp	Kyn	Trp	Kyn
pH 2.0	10.5	11.0	102.8	100.5
pH 5.5	9.8	10.3	96.2	94.0
pH 7.4	9.9	10.9	96.5	99.9
pH 8.8	10.6	10.5	103.8	96.4
pH 12.0	10.1	5.3	99.1	48.8

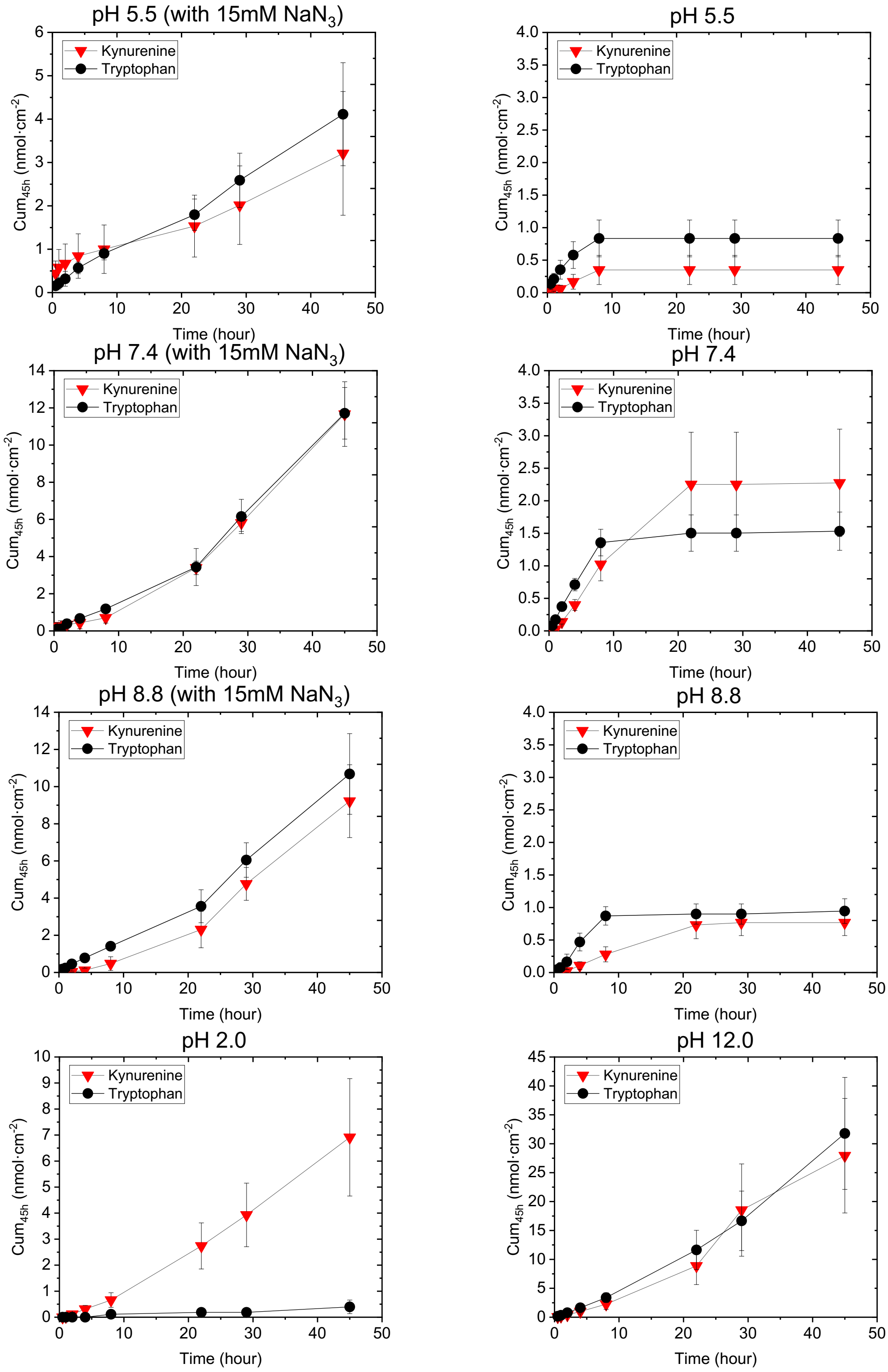

Figure S1. Left panel shoe permeation profiles (nmol cm^{-2}, mean ± SEM) of tryptophan (Trp) and kynurenine (Kyn) in the presence of the antimicrobial agent sodium azide (15 mM NaN_3) in both donor and receptor chambers (n=6). Right panel show the corresponding results without presence of NaN_3 (n=3). Results obtained at pH 2.0 and pH 12.0 were without NaN_3. The pH of donor and receptor chamber were the same. Trp and Kyn concentrations in the lower Franz cells chamber were 1 mM.

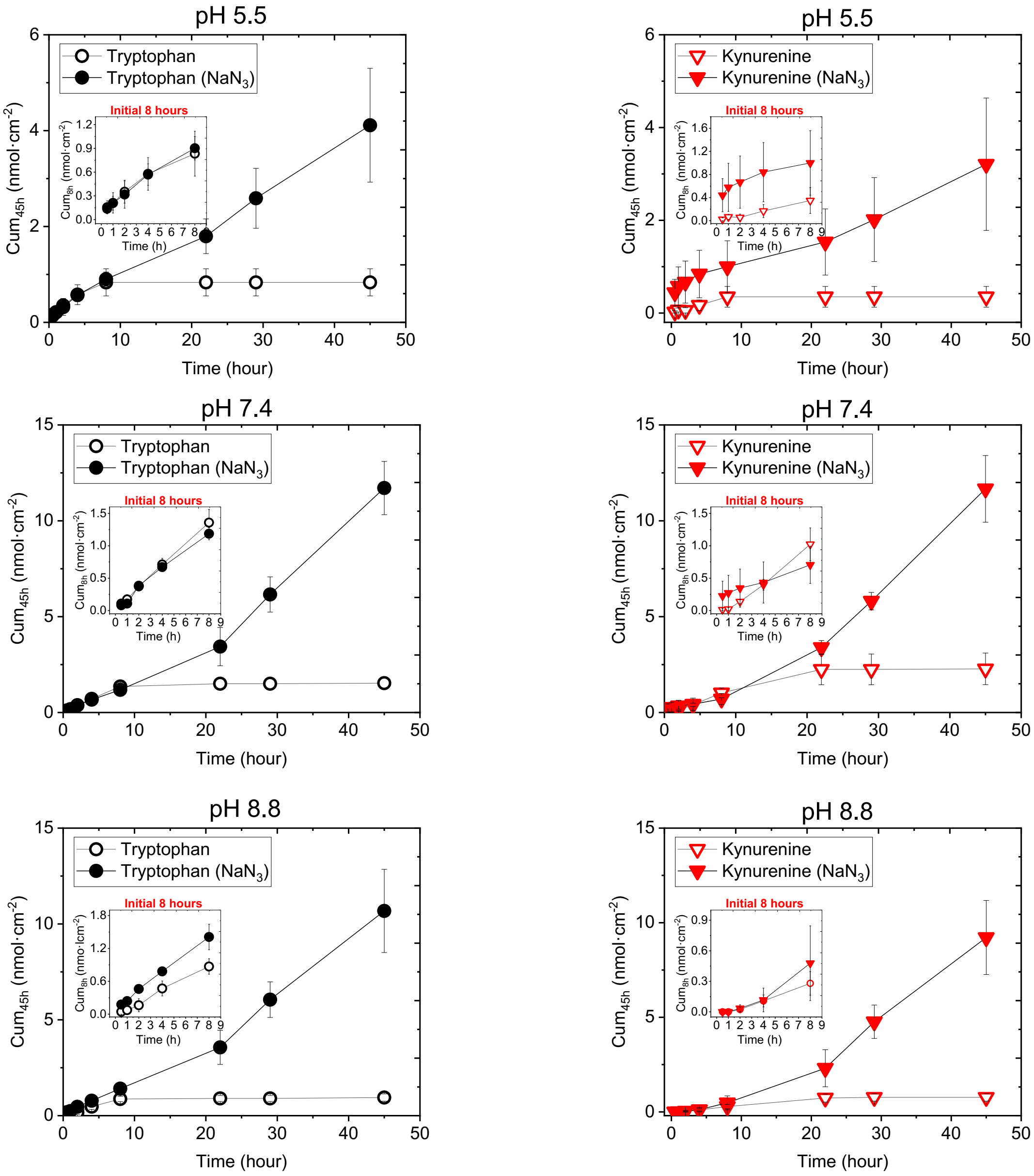

Figure S2. For a clear comparison, the results presented in Figure S1 is replotted by presenting results obtained with 15 mM NaN_3 present in both donor and receptor chambers (n=6) and without (n=3) in the same plot for pH 5.5 (top), 7.4 (middle), and 8.8 (bottom). The permeation profiles (nmol cm^{-2}, mean ± SEM) of tryptophan (Trp) is shown in the left panel and the permeation profiles of kynurenine (Kyn) is found in the right panel. The pH of donor and receptor chamber were the same. Trp and Kyn concentrations in the lower Franz cells chamber were 1 mM.

Calculation of tryptophan and kynurenine permeability constants based on the four permeation pathways theory

The theoretical flux of tryptophan (Trp) and kynurenine (Kyn) can be expressed according to the four-pathway model by the following equation:

$$flux\left[\frac{nmol}{cm^2 s}\right] = (K_p^{fv} + K_p^{lateral} + K_p^{shunt} + K_p^{pore})\left[\frac{cm}{s}\right] \times C\left[\frac{nmol}{cm^3}\right] \qquad \text{Eq. S1}$$

where K_p^{fv}, $K_p^{lateral}$, K_p^{shunt}, and K_p^{pore} are the permeability constants accounting for solute diffusion via free-volume diffusion through the lipid lamellar matrix, lateral diffusion along lipid multilamellar layers, diffusion through shunt pathways, and diffusion through aqueous pores (defects) of the lipid multilamellar matrix.[8] The concentration C refers to the permeant (Trp or Kyn), which in this work was 1000 nmol cm^{-3} for all experiments. The equations describing the four permeability constants are presented below.[8] For the calculations of these constants, we used the physicochemical data summarized in the Table S4 for Trp and Kyn. The obtained results from the theoretical calculations are compiled in Table S7.

Table S4. Physicochemical data of tryptophan and kynurenine used for estimation of the theoretical permeability constants. The values of the distribution coefficient (log D) were obtained from the Chemicalize software (ChemAxon).

Permeant	van der Waals radius, $r_{vdW,p}$ (Å)	Hydrodynamic radius, $r_{h,p}$ (Å)	logD
L-tryptophan (MW 204.23 g/mol) 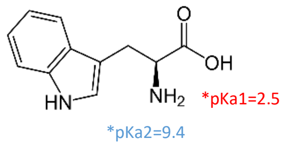	3.54	4.33	-1.5 (pH 2.0)<br>-1.1 (pH 5.5)<br>-1.1 (pH 7.4)<br>-1.1 (pH 8.8)<br>-2.3 (pH 12.0)
L-kynurenine (MW 208.22 g/mol) 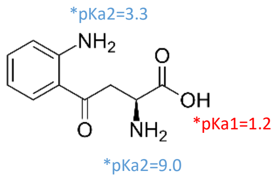	3.56	4.36	-3.1 (pH 2.0)<br>-1.9 (pH 5.5)<br>-1.9 (pH 7.4)<br>-2.1 (pH 8.8)<br>-3.2 (pH 12.0)

Free-volume diffusion (K_p^{fv})

Free volume diffusion (K_p^{fv}) is dominated by transport of small (M_w < 400-500 Da) and hydrophobic ($K_{o/w}$ > 1) permeants. It is hypothesized that this route of permeation occurs via density fluctuations in the lipid chains that open up free volume pockets available for diffusion.[8] The permeability constant characterizing (K_p^{fv}) is given by:

$$K_p^{fv}\left[\frac{cm}{s}\right] = \frac{D_b\left[\frac{cm^2}{s}\right] \times K_b}{\tau^* \times d_{SC}[cm]} = \frac{2 \times 10^{-5} \times exp(-0.46 \times r_{vdW,p}{}^2)\left[\frac{cm^2}{s}\right] \times K_{ow}^{0.7}}{3.6[cm]} \qquad \text{Eq. S2}$$

where D_b represents the diffusion coefficient of the permeant in a lipid bilayer, $K_b = K_{ow}^{0.7}$ is the solute partition coefficient, τ^* is the effective tortuosity factor, d_{SC} is the thickness of SC (15×10^{-4} cm), and $r_{vdW,p}$ is the van der Waals radius of the permeant.[8] The product of the parameters $\tau \times d_{SC}$ has previously been reported to be equal to 3.6 cm [9] and therefore used in this work, as well as in previous studies.[8,10] The parameter $r_{vdW,p}$ of Trp and Kyn was estimated from the molecular weight (see Table S1) based on the following relationship $\frac{4}{3}\pi r^3 = 0.9087 \times M_w\ [Da]$ [8]. Note that $K_{o/w}$ is estimated to be equal to the distribution coefficient (D, see Table S1).

Lateral diffusion ($K_p^{lateral}$)

Lateral diffusion ($K_p^{lateral}$) is mainly relevant for large hydrophobic solutes ($M_w > 500$ Da) with similar molecular weights as the lipid species of the extracellular lipid lamellar matrix of SC. These molecules are likely to partition inside the lipid matrix, but unable to diffuse via free volume pockets as described above. Thus, this type of diffusion process is expected to be on the same order of magnitude as the lateral diffusion coefficient of the SC lipid species (3×10^{-9} cm^2/s) and can to a first approximation be calculated by the following equation.[8]

$$K_p^{lateral}\left[\frac{cm}{s}\right] = \frac{D_b^{lateral}\left[\frac{cm^2}{s}\right] \times K_b}{\tau^* \times d_{SC}[cm]} = \frac{3 \times 10^{-9}\left[\frac{cm^2}{s}\right] \times K_{ow}^{0.7}}{3.6[cm]} \qquad \text{Eq. S3}$$

where $D_b^{lateral}$ is the lateral diffusion coefficient of a lipid in the SC lipid lamellar matrix and $K_b = K_{ow}^{0.7}$ is the solute partition coefficient in the lipid matrix (τ^* and d_{SC} have the same meaning as in Eq. S2).[8]

Shunt pathway (K_p^{shunt})

The shunt pathway (K_p^{shunt}) represents permeation through hair follicles and sweat ducts and is primarily important for hydrophilic molecules with high molecular weights. Diffusion based on K_p^{shunt} is described by the following equation:

$$K_p^{shunt}\left[\frac{cm}{s}\right] = \frac{\emptyset_s D_s}{L_{shunt}} = 2 \times 10^{-9}\left[\frac{cm}{s}\right] \qquad \text{Eq. S4}$$

Where $\emptyset_s$ is area fraction covered by hair follicles and sweat ducts (10^{-4} cm^2/cm^2), D_s is diffusion coefficient of the solute in a shunt (1×10^{-6} cm^2/s) and L_{shunt} is the length of a shunt (500 µm).[8] Strictly speaking, the value of D_s decreases with increasing molecular weight according to Stokes–

Einstein equation. However, as previously suggested, the contribution of shunts is dominant only for hydrophilic molecules with $M_w > 100$ kDa, and thus expected to be of minor importance for Trp and Kyn. To a first approximation, the contribution of shunts to the skin permeability was estimated to be constant and equal to $K_p^{shunt} = 2\times10^{-9}$ cm/s, independent of molecular size and property.[8]

Porous pathway (imperfections in the multilamellar lipid matrix, K_p^{pore})

The aqueous pore pathway (K_p^{pore}) is suggested to represent a preferential pathway across SC for small hydrophilic molecules ($K_{o/w} < 1$ and $M_w < 500$ Da).[11] The rationale for the existence of "pores" in the SC is based on highly dynamical imperfections in the multilamellar lipid matrix surrounding the corneocytes in the SC, which support water-filled pores. These imperfections may manifest themselves as, for example, separation of grain boundaries, vacancies in the lipid lattice leading to multimolecular voids, or defects created by steric constraints due to the highly curved geometry of the lipid matrix rounding the corneocytes. The overall effect of these defects is conceptualized as hydrophilic short-lived channels with a pore radius equal to r where diffusion of water-soluble solutes across otherwise poorly permeable lipid regions can occur.[8,10] Diffusion via the pore pathway is based on several assumptions, which are discussed in detail elsewhere.[8,10-12] A general expression of the permeability coefficient K_p^{pore} is given by the following equation:

$$K_p^{pore}\left[\frac{cm}{s}\right] = \frac{\varepsilon \times D_p\left[\frac{cm^2}{s}\right]}{\tau_p \times d_{SC}[cm]} = \frac{\varepsilon \times D_p^{\infty}\left[\frac{cm^2}{s}\right] \times H(\lambda_p)}{\tau_p \times d_{SC}[cm]} \qquad \text{Eq. S5}$$

In the equation above, ε is the SC porosity, τ_p is tortuosity of the SC, d_{SC} is the SC thickness, and D_p is the solute diffusion coefficient in the aqueous pore.[8,11] As shown, D_p is a function of both the permeant and structural properties of the SC membrane, and can be represented as a product of the diffusion coefficient of the permeant at infinite dilution D_p^{∞} and the hindrance factor $H(\lambda_p)$. Here, λ_p is the ratio of the hydrodynamic radius of the permeant $r_{h,p}$ (see Table S1) and the effective pore radius of the SC r (i.e., $\lambda_p = r_{h,p}/r$). For $\lambda_p < 0.4$, the diffusion hindrance factor, $H(\lambda_p)$, can be calculated as follows:[11,13]

$$H(\lambda_p) = (1 - \lambda_p)^2(1 - 2.104\lambda_p + 2.09\lambda_p^3 - 0.95\lambda_p^5) \qquad \text{Eq. S6}$$

As proposed by Tang et al.,[12] an equation similar to Eq. S5 can be written for the ions that carries the passive current during the impedance measurements. For clarity, this equation is presented below:

$$K_{ion}^{pore}\left[\frac{cm}{s}\right] = \frac{\varepsilon \times D_{ion}\left[\frac{cm^2}{s}\right]}{\tau_{ion} \times d_{SC}[cm]} = \frac{\varepsilon \times D_{ion}^{\infty}\left[\frac{cm^2}{s}\right] \times H(\lambda_{ion})}{\tau_{ion} \times d_{SC}[cm]} \qquad \text{Eq. S7}$$

In Eq. S7, $\lambda_{ion} = r_{h,ion}/r$ where $r_{h,ion}$ is the hydrodynamic radius of the ion (a value equal to 2.2 Å was used for $r_{h,ion}$), while all other parameters have the same meaning as in Eq. S4, but for the ion instead of the permeant. By taking the ratio of Eq. S5 and Eq. S7 we can express the permeability of the hydrophilic permeant as a linear function of the passive ion permeability according to:

$$K_p^{pore}\left[\frac{cm}{s}\right] = K_{ion}^{pore}\left[\frac{cm}{s}\right] \times \frac{D_p^{\infty}\left[\frac{cm^2}{s}\right] \times H(\lambda_p)}{D_{ion}^{\infty}\left[\frac{cm^2}{s}\right] \times H(\lambda_{ion})} \qquad \text{Eq. S8}$$

The diffusion coefficient at infinite ion dilution, D_{ion}^{∞}, is related to the absolute ion mobility u_{abs} via the Einstein relation $D_{ion}^{\infty} = kTu_{abs}$, which can be related to the conductivity of the electrolyte solution σ_{sol}.[12] Further, by assuming that the cations and anions have the same mobilities, the following expression can be derived for D_{ion}^{∞}:[12]

$$D_{ion}^{\infty}\left[\frac{cm^2}{s}\right] = \frac{k\left[\frac{J}{K}\right] \times T[K]}{2 \times z^2 \times F\left[\frac{C}{mol}\right] \times c_{ion}\left[\frac{mol}{cm^3}\right] \times e_0[C]} \times \sigma_{sol}[Ohm^{-1} \times cm^{-2}] \qquad \text{Eq. S9}$$

Next, by defining the skin conductivity σ_{SC} on basis of the ion conductivity σ_{sol}, in a similar manner as compared to the permeability coefficient of a permeant (e.g. see Eq. S5), the following equation can be derived:[12]

$$\sigma_{SC}[Ohm^{-1} \times cm^{-2}] = \frac{\varepsilon}{\tau} \times \sigma_{sol}[Ohm^{-1} \times cm^{-2}] \times H(\lambda_{ion}) \qquad \text{Eq. S10}$$

Eq. S10 can be rewritten and inserted in Eq. S9, after which one obtains the following expression:[12]

$$D_{ion}^{\infty}\left[\frac{cm^2}{s}\right] = \frac{k\left[\frac{J}{K}\right] \times T[K]}{2 \times z^2 \times F\left[\frac{C}{mol}\right] \times c_{ion}\left[\frac{mol}{cm^3}\right] \times e_0[C]} \times \frac{\sigma_{SC}[Ohm^{-1} \times cm^{-2}] \times \tau_{ion}}{\varepsilon \times H(\lambda_{ion})} \qquad \text{Eq. S11}$$

The expression of D_{ion}^{∞} (Eq. S11) can now be inserted into Eq. S7 to obtain the following equation for K_{ion}^{pore}:[12]

$$K_{ion}^{pore}\left[\frac{cm}{s}\right] = \frac{k\left[\frac{J}{K}\right] \times T[K]}{2 \times z^2 \times F\left[\frac{C}{mol}\right] \times c_{ion}\left[\frac{mol}{cm^3}\right] \times e_0[C]} \times \frac{\sigma_{SC}[Ohm^{-1} \times cm^{-2}]}{d_{SC}[cm]} \qquad \text{Eq. S12}$$

where σ_{SC} can be defined in terms of the skin membrane resistance R_{mem}. The latter parameter can easily be determined experimentally and related to σ_{SC} according to:[12]

$$\sigma_{SC} = \frac{d_{SC}[cm]}{R_{mem}[Ohm \times cm^2]} \qquad \text{Eq. S13}$$

For clarity, we insert Eq. S13 into Eq. S12 and obtain the following expression:[12]

$$K_{ion}^{pore}\left[\frac{cm}{s}\right] = C_A\left[\frac{Ohm \times\ cm^3}{s}\right] \times \frac{1}{R_{mem}[Ohm \times cm^2]} \qquad \text{Eq. S14}$$

where $C_A$is defined as:

$$C_A\left[\frac{Ohm \times\ cm^3}{s}\right] = \frac{k\left[\frac{J}{K}\right] \times T[K]}{2 \times z^2 \times F\left[\frac{C}{mol}\right] \times c_{ion}\left[\frac{mol}{cm^3}\right] \times e_0[C]} \qquad \text{Eq. S15}$$

A unit analysis of the C_A yields:

$$C_A = \frac{J\ \times cm3}{C^2} = \frac{kg \times cm^2 \times cm^3}{s^2 \times A^2 \times s^2} = \left[\frac{kg \times\ m^2}{s^3\ \times A^2} = Ohm\right] = \frac{Ohm \times\ cm^3}{s} \qquad \text{Eq. S16}$$

which is consistent with:

$$C_A = K_{ion}^{pore}[\frac{cm}{s}] \times R_{mem}[Ohm \times cm^2] \qquad \text{Eq. S17}$$

The expression of K_{ion}^{pore} (Eq. S14) can now be inserted in Eq. S8:

$$K_p^{pore}\left[\frac{cm}{s}\right] = C_A\left[\frac{Ohm \times\ cm^3}{s}\right] \times \frac{1}{R_{mem}[Ohm \times cm^2]} \times \frac{D_p^{\infty}\left[\frac{cm^2}{s}\right] \times H(\lambda_p)}{D_{ion}^{\infty}\left[\frac{cm^2}{s}\right] \times H(\lambda_{ion})} \qquad \text{Eq. S18}$$

By defining C_B according to following:[12]

$$C_B\left[\frac{Ohm \times\ cm^3}{s}\right] = C_A\left[\frac{Ohm \times\ cm^3}{s}\right] \times \frac{D_p^{\infty}\left[\frac{cm^2}{s}\right] \times H(\lambda_p)}{D_{ion}^{\infty}\left[\frac{cm^2}{s}\right] \times H(\lambda_{ion})} \qquad \text{Eq. S19}$$

and inserting C_B into Eq. S18, we obtain:

$$K_p^{pore}\left[\frac{cm}{s}\right] = C_B\left[\frac{Ohm \times\ cm^3}{s}\right] \times \frac{1}{R_{mem}[Ohm \times cm^2]} \qquad \text{Eq. S20}$$

By taking the logarithm of Eq. S20 one obtains:

$$\log_{10}[K_p^{pore}] = -1 \times \log_{10}[R_{mem}] + \log_{10}[C_B] \qquad \text{Eq. S21}$$

Eq. S21 can be used to evaluate the individual permeability data, together with the corresponding individual membrane resistance (R_m) data, to obtain the individual values of the C_B.[12] This analysis assumes that a plot of $\log_{10}[K_{ion}^{pore}]$ versus $\log_{10}[R_m]$ follows a linear dependence with the slope equal to -1 and the intercept equal to $\log_{10}[C_B]$.

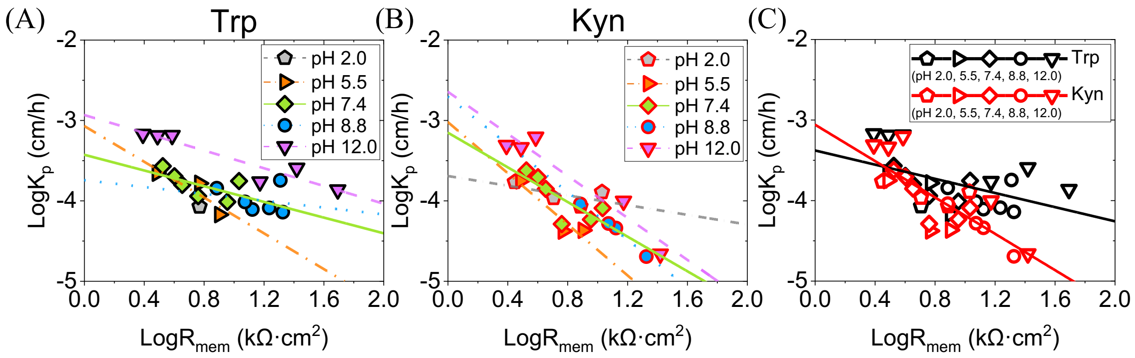

Figure S3. Logarithmic plots of experimentally determined skin permeability coefficients (K_p) versus skin membrane resistance values (R_{mem}) for (A) Trp, (B) Kyn, and (C) both permeants. In (A) and (B), data sets obtained at specific pH values are treated separately (i.e., the lines correspond to linear fits of the data at different pH values). In (C), the experimental values are combined and treated as one data set for Trp or Kyn, respectively. All values from the linear regression analysis are compiled in Table S5. The permeability coefficients (K_p) were obtained from the cumulative amount permeated after 8h and the initial skin membrane resistance (R_{mem}).

Table S5. Summary of data from the regression analysis from plots of $\log_{10}[K_p^{pore}]$ versus $\log_{10}[R_{mem}]$, see Fig. S3. The intercepts (where $\log_{10}[R_{mem}] = 0$) represent $\log_{10}[C_B]$ in Eq. S21. The coefficients of determination (r^2) referrers to the regression lines in Fig. S3. Note that the slopes are theoretically expected to be -1, which is sometimes clearly not the case. Therefore, each data point in Fig. S3 was extrapolated using the ideal slope value of -1 to obtain individual values of C_B according to Eq. S21. The values of C_B were then averaged for each pH value and compiled in Table S6. This procedure follows from previous work.[12]

Condition	Permeant	Slope	Intercept	r^2	n
pH 2.0	Trp	N.A.	N.A.	N.A.	1
	Kyn	-0.3076	-3.6908	0.3489	4
pH 5.5	Trp	-1.1125	-3.0693	0.7774	3
	Kyn	-1.5890	-3.0210	0.8936	3
pH 7.4	Trp	-0.4883	-3.4269	0.3695	6
	Kyn	-1.0774	-3.1522	0.6107	6
pH 8.8	Trp	-0.2128	-3.7428	0.0495	6
	Kyn	-1.4814	-27062	0.9903	4
pH 12.0	Trp	-0.5515	-2.9326	0.8918	6
	Kyn	-1.3072	-2.6435	0.9187	5
pH 2.0, 5.5, 7.4, 8.8, 12.0	Trp	-0.4398	-3.3782	0.2650	22
	Kyn	-1.1287	-3.0584	0.6611	22

Once the individual values of C_B (i.e., the intercepts) are obtained, it is possible to calculate the ratio $\frac{H(\lambda_p)}{H(\lambda_{ion})}$ according Eq. S22:

$$C_B\left[\frac{Ohm \times cm^3}{s}\right] = C_C\left[\frac{Ohm \times cm^3}{s}\right] \times \frac{H(\lambda_p)}{H(\lambda_{ion})} \qquad \text{Eq. S22}$$

Note that C_C in Eq. S22 only contains parameters with known values and can be expressed according to:

$$C_C\left[\frac{Ohm \times cm^3}{s}\right] = \frac{k\left[\frac{J}{K}\right] \times T[K]}{2 \times z^2 \times F\left[\frac{C}{mol}\right] \times c_{ion}\left[\frac{mol}{cm^3}\right] \times e_0[C]} \times \frac{D_p^\infty\left[\frac{cm^2}{s}\right]}{D_{ion}^\infty\left[\frac{cm^2}{s}\right]} \qquad \text{Eq. S23}$$

In these calculations, the mean value of individual C_B values, from each pH level, was used to obtain the corresponding value of the ratio $\frac{H(\lambda_p)}{H(\lambda_{ion})}$. In other words, the values of C_B obtained from the linear regression analysis was not used, which is in accordance to previous work.[12] Next, Eq. S22 was solved iteratively for a solution of the pore radius r for each pH value (see Table S6). For clarity, Eq. S22 can be rewritten in the actual form that was iteratively evaluated by the software Wolfram Alpha for a solution of the pore radius r:

$$\frac{C_B}{C_C} = \frac{H(\lambda_p)}{H(\lambda_{ion})} = \frac{\left[1-\frac{r_p}{r}\right]^2 \times \left[1 - 2.104 \times \frac{r_p}{r} + 2.09 \times \left(\frac{r_p}{r}\right)^3 - 0.95 \times \left(\frac{r_p}{r}\right)^5\right]}{\left[1-\frac{r_{ion}}{r}\right]^2 \times \left[1 - 2.104 \times \frac{r_{ion}}{r} + 2.09 \times \left(\frac{r_{ion}}{r}\right)^3 - 0.95 \times \left(\frac{r_{ion}}{r}\right)^5\right]} \qquad \text{Eq. S24}$$

As a control, $\lambda_p = r_{h,p}/r$ was calculated to confirm that $\lambda_p < 0.4$, which is the criterion for Eq. S6 to be valid. As shown in Table S6, when λ_p was determined based on values of C_B from each pH value, this criterion was not always fulfilled. This is likely due to the fact that the biological variation of the skin membranes become more pronounced in small data sets, while larger data sets tend to limit the influence of biological variation. Therefore, to resolve the problem when $\lambda_p > 0.4$ (or when r could not be determined, which was the case at pH 12.0), it was decided to use a value of λ_p based on a wider data set, corresponding to the mean value of C_B obtained at pH values of 2.0, 5.5, 7.4, 8.8, and 12.0. This strategy was used in the three cases marked with * in Table S6. Finally, the permeability coefficient K_p^{pore} was calculated according to Eq. S20 by employing the initial values of R_{mem} from each skin membrane; the mean value of K_p^{pore} from each pH level is compiled in Table S7.

For comparison, K_p^{pore} was also calculated according to Eq. S5. However, this equation always generated significantly higher values, most likely due to the great uncertainty of the estimated values of the input parameters. In addition, Eq. S5 only relies on the diffusion hindrance factor for the

permeant $H(\lambda_p)$, while Eq. S20 uses the ratio of the hindrance factor for the permeant $H(\lambda_p)$ and the ion $H(\lambda_{ion})$. For these reasons, we only consider the results obtained with Eq. S20 from now on.

Table S6. Compilation of data of C_B, obtained from mean values of individual intercepts, (kOhm cm³ h^{-1}), pore radius r (Å), ratio between the hydrodynamic radius of the permeant and the pore radius λ_p, the hindrance factor $H(\lambda_p)$. *Note that if $\lambda_p > 0.4$ (or when r could not be determined) the hindrance factor $H(\lambda_p)$ is marked as N/A, which indicates that the theoretical approach is not applicable.

Condition	Analyte	$C_B \times 10^{-4}$	r	λ_p	$H(\lambda_p)$
pH 2.0	Trp	4.32	9.98	0.43*	N.A.
	Kyn	6.99	13.22	0.33	0.17
pH 5.5	Trp	7.08	13.07	0.33	0.17
	Kyn	3.59	9.30	0.47*	N.A.
pH 7.4	Trp	9.10	16.34	0.27	0.26
	Kyn	6.16	12.24	0.36	0.14
pH 8.8	Trp	14.75	40.00	0.11	0.62
	Kyn	5.80	11.78	0.37	0.13
pH 12.0	Trp	31.43	N.A.	N.A.*	N.A.
	Kyn	12.80	27.70	0.16	0.48
pH 2.0, 5.5, 7.4, 8.8, 12.0	Trp	13.60	31.35	0.14	0.53
	Kyn	6.84	12.97	0.34	0.16

Theoretical contribution of each of the four permeation pathways

Finally, the theoretical permeability constants K_p^{fv}, $K_p^{lateral}$, K_p^{shunt}, and K_p^{pore} were calculated for each permeation pathway for Trp and Kyn at different pH values according to Eq. S2, S3, S4, and S20, respectively. All data are compiled in Table S7.

Table S7. Contribution of each of the four permeability constants to the total permeability constant (K_p) for tryptophan (Trp) and kynurenine (Kyn) based on calculations following the four permeation pathways theory. Values marked with * indicate that $\lambda_p > 0.4$ (or that r could not be determined). In these cases, K_p^{pore} was estimated from the mean value of C_B obtained at pH values of 2.0, 5.5, 7.4, 8.8, and 12.0 (see Table S6).

pH	Permeability constants for each pathway and their sum, K_p	K_p × 10^-8 (cm/s)		Contribution to K_p (%)	
		Trp	Kyn	Trp	Kyn
2.0	Free volume diffusion, K_p^{fv}	0.16	0.01	2.58	0.35
	Lateral diffusion, $K_p^{lateral}$	0.01	0.00	0.12	0.02
	Shunt pathway, K_p^{shunt}	0.20	0.20	3.33	6.42
	Pore pathway, K_p^{pore}	5.65*	2.90	93.96	93.21
	K_p	6.01	3.11	100	100
5.5	Free volume diffusion, K_p^{fv}	0.30	0.08	8.64	2.47
	Lateral diffusion, $K_p^{lateral}$	0.01	0.00	0.41	0.13
	Shunt pathway, K_p^{shunt}	0.20	0.20	5.84	6.46
	Pore pathway, K_p^{pore}	2.91	2.82*	85.10	90.96
	K_p	3.42	3.10	100	100
7.4	Free volume diffusion, K_p^{fv}	0.30	0.08	5.53	2.15
	Lateral diffusion, $K_p^{lateral}$	0.01	0.00	0.26	0.11
	Shunt pathway, K_p^{shunt}	0.20	0.20	3.74	5.63
	Pore pathway, K_p^{pore}	4.84	3.28	90.47	92.12
	K_p	5.35	3.56	100	100
8.8	Free volume diffusion, K_p^{fv}	0.30	0.06	8.34	3.81
	Lateral diffusion, $K_p^{lateral}$	0.01	0.00	0.40	0.19
	Shunt pathway, K_p^{shunt}	0.20	0.20	5.63	13.76
	Pore pathway, K_p^{pore}	3.04	1.20	85.63	82.24
	K_p	3.55	1.45	100	100
12.0	Free volume diffusion, K_p^{fv}	0.04	0.01	2.58	0.35
	Lateral diffusion, $K_p^{lateral}$	0.00	0.00	0.12	0.02
	Shunt pathway, K_p^{shunt}	0.20	0.20	3.33	6.42
	Pore pathway, K_p^{pore}	7.03*	6.61	93.96	93.21
	K_p	7.27	6.82	100	100

References

1 Weinlich, G., Murr, C., Richardsen, L., Winkler, C. & Fuchs, D. Decreased serum tryptophan concentration predicts poor prognosis in malignant melanoma patients. *Dermatology* **214**, 8-14, (2006).

2 Barjandi, G. *et al.* Plasma tryptophan and kynurenine in females with temporomandibular disorders and fibromyalgia – An exploratory pilot study. *Journal of Oral Rehabilitation*, 1-8, (2019).

3 Wu, Y. *et al.* Kynurenine pathway changes in late-life depression. *Journal of Affective Disorders* **235**, 76-81, (2018).

4 Lyon, D. E., Walter, J. M., Starkweather, A. R., Schubert, C. M. & McCain, N. L. Tryptophan degradation in women with breast cancer: A pilot study. *BMC Research Notes* **4**, (2011).

5 Yu, J. *et al.* A tryptophan metabolite of the skin microbiota attenuates inflammation in patients with atopic dermatitis through the aryl hydrocarbon receptor. *Journal of Allergy and Clinical Immunology* **143**, 2108-2119.e2112, (2019).

6 Li, H. *et al.* Metabolomic adaptations and correlates of survival to immune checkpoint blockade. *Nature Communications* **10**, 3-8, (2019).

7 Triplett, T. A. *et al.* Reversal of indoleamine 2,3-dioxygenase–Mediated cancer immune suppression by systemic kynurenine depletion with a therapeutic enzyme. *Nature Biotechnology* **36**, (2018).

8 Mitragotri, S. Modeling skin permeability to hydrophilic and hydrophobic solutes based on four permeation pathways. *Journal of Controlled Release* **86**, 69-92, (2003).

9 Johnson, M. E., Blankschtein, D. & Langer, R. Evaluation of solute permeation through the stratum corneum: Lateral bilayer diffusion as the primary transport mechanism. *Journal of pharmaceutical sciences* **86**, 1162-1172, (1997).

10 Tezel, A., Sens, A. & Mitragotri, S. Description of transdermal transport of hydrophilic solutes during low-frequency sonophoresis based on a modified porous pathway model. *Journal of pharmaceutical sciences* **92**, 381-393, (2003).

11 Peck, K. D., Ghanem, A.-H. & Higuchi, W. I. Hindered diffusion of polar molecules through and effective pore radii estimates of intact and ethanol treated human epidermal membrane. *Pharmaceutical Research* **11**, 1306-1314, (1994).

12 Tang, H., Mitragotri, S., Blankschtein, D. & Langer, R. Theoretical description of transdermal transport of hydrophilic permeants: Application to low-frequency sonophoresis. *Journal of pharmaceutical sciences* **90**, 545-568, (2001).

13 Deen, W. M. Hindered transport of large molecules in liquid-filled pores. *AIChE Journal* **33**, 1409-1425, (1987).
